# Medium-sized exotic prey create novel food webs: the case of predators and scavengers consuming lagomorphs

**DOI:** 10.7717/peerj.2273

**Published:** 2016-07-27

**Authors:** Facundo Barbar, Fernando Hiraldo, Sergio A. Lambertucci

**Affiliations:** 1 Grupo de Biología de la Conservación, Ecotono Laboratory, INIBIOMA—CONICET (Universidad Nacional del Comahue), San Carlos de Bariloche, Río Negro, Argentina; 2 Departmento de Biología de la Conservación, Estación Biológica Doñana—CSIC España, Sevilla, España

**Keywords:** Lagomorpha, Exotic prey, Oceania, Food webs, South America, Top predators

## Abstract

Food web interactions are key to community structure. The introduction of species can be seen as an uncontrolled experiment of the addition of species. Introduced species lead to multiple changes, frequently threatening the native biodiversity. However, little is known about their direct effect on the upper level of the food web. In this study we review empirical data on the predator–prey relationship between the introduced lagomorphs and their consumers, and use meta-analytical tools to quantify the strength of their interactions. We expect that exotic lagomorphs will destabilize food webs, affect ecological processes and compromise the conservation of the invaded regions. We found 156 studies on the diet of 43 species of predators that consume lagomorphs as exotic preys in South America and Oceania. We found an average exotic lagomorphs-predator link of 20% which indicates a strong interaction, given that the average for the strongest links with native prey (when lagomorphs are not included in the predator diet) is about 24%. Additionally, this last link decreases to 17% when lagomorphs are present. When lagomorphs arrive in a new environment they may become the most important resource for predators, producing an unstable equilibrium in the novel food web. Any disruption of this interaction could have catastrophic consequences for the native diversity by directly impacting predators or indirectly impacting native preys by apparent competition. Eradication or any change in their abundances should be carefully considered in conservation actions since those will have great impacts on predator populations and ultimately in the whole communities.

## Introduction

Food web interactions are key in community structure and provide useful information about how an ecosystem works (Krebs et al., 1999; Ings et al., 2009). The strength of the links within a web plays an important role in its stability, where diverse communities with weaker links tend to be more stable than those that are less diverse and with stronger links (Berlow, 1999; Berlow et al., 2004). In this sense, the introduction of species can be seen as a uncontrolled experiment of the addition of species (McCann, 2000). As exotic species grow in abundance they generally become a new resource subsidy (Huxel & McCann, 1998; Jefferies, 2000; Tablado et al., 2010). This may lead to the formation of a strong food web link between these novel species and the consumers, that will depend on the availability of the former and on the consumers’ ability to exploit them as a resource (Rodriguez, 2006).

Introduced species generate several changes in an invaded ecosystem, frequently threatening the native biodiversity through the alteration of both ecological functions and food webs, and the dynamics of native populations by predation or competition (Vitousek et al., 1996; Simberloff et al., 2012). However, in some cases introduced species might be beneficial for some native species through facilitation and resource subsidy, among other mechanisms (Rodriguez, 2006; Schlaepfer, Sax & Olden, 2011; Speziale & Lambertucci, 2013). Of these exotic species, introduced terrestrial herbivores are known to compete directly with native herbivores, generating changes in the plant community, facilitating plant invasions and even causing direct or indirect animal extinctions via apparent competition (Courchamp, Langlais & Sugihara, 2000; Holmgren, 2002; Clavero & García-Berthou, 2005; Best & Arcese, 2009). However, studies that directly measure the effect of introduced terrestrial herbivores on the higher trophic level of the food webs are scarce, aside from some studies on invasive freshwater and insect preys (Barber, Marquis & Tori, 2008; Carlsson, Sarnelle & Strayer, 2009; Tablado et al., 2010; Carlsson et al., 2011; Dijkstra, Lambert & Harris, 2012), even though these predator–prey interactions might be affecting the whole community (Schmitz, Hambäck & Beckerman, 2000; Estes et al., 2011).

The order Lagomorpha was originally distributed worldwide, with the exception of Antarctica, southern South America and Oceania, and includes more than ninety species (Alves, Ferrand & Hackländer, 2008). Of the six Lagomorph species introduced in several continents, the European hare (*Lepus europaeus*) and European rabbit (*Oryctolagus cuniculus*) are important invasive species, given their abundance and distribution (Alves, Ferrand & Hackländer, 2008; IUCN, 2015). Those species have invaded southern South America and Oceania, where they do not have an ecological equivalent (Dawson & Ellis, 1994; Robley, Short & Bradley, 2001; Lees & Bell, 2008). They have dispersed rapidly since their introduction (between 10–20 km/year and up to 100 km/year in some cases (Grigera & Rapoport, 1983; Stodart & Parer, 1988)). Because of their size and abundance they are consumed by several predator species, being some of them considered as keystone species in their native range (Delibes & Hiraldo, 1981; Delibes-Mateos et al., 2011).

Predators and scavengers (hereafter referred to as predators) such as birds of prey, felids and foxes, among others, consume these preys in both their native and exotic range (i.e., Tjernberg, 1981; Gil-Sánchez, Ballesteros-Duperón & Bueno-Segura, 2006; Lambertucci et al., 2009; Olsen et al., 2010; Zanón Martínez et al., 2012). These predators can be used as indicator species as they have large home ranges, are charismatic, conspicuous, well studied and susceptible to environmental changes in various ways (Sergio et al., 2008) to assess some ecological traits of invasion and its consequences in terms of stability and conservation of the invaded communities in general (Carroll, Noss & Paquet, 2001; Barber, Marquis & Tori, 2008). Predators plastic feeding behavior and the number of studies on their diet, make them a suitable model to study how lagomorph interact with the upper levels of an invaded food web (Dupuy, Giraudoux & Delattre, 2009; Tablado et al., 2010).

In this study, we review empirical data on the predator–prey relationship (dietary links) between introduced lagomorphs and native and exotic consumers, to quantify the strength of the link between these two trophic levels. For this purpose, we use meta-analytical tools to quantify the consumption frequency (occurrence of a particular prey item over the total, sensu Berlow et al., 2004) as a measure of the interaction strength between these two trophic levels. Our hypothesis is that due to their high abundance and availability, lagomorphs will enter the trophic network modifying the predator–prey relationships, which may affect ecological processes (e.g., consumption rates, energy flux, competition) and the conservation of the invaded regions. We expect that: (1) predators will form strong interaction links with the exotic preys, which will be equivalent to the interactions with the most consumed native preys, and (2) that the interactions predator—native prey will be weaker when exotic lagomorphs appear in the diet.

## Methods

### Bibliographic search

We used Scopus and Google Scholar search engines to find studies detailing predators diet in South America and Oceania, the two continents where lagomorphs (in particular *L. europaeus* and *O. cuniculus*) were introduced and widely distributed. We conducted the search individually for each predator species based on information about their body size and foraging behavior that could include lagomorphs in their diet consumed either as preys or carrion (i.e., we left out of the search insectivore or light weight predators; <0.2 kg. for birds and mammals). We intentionally left out of the search domestic species as feral cats and dogs. Our search was conducted by predators instead of by prey, because we observed from previous searches on the prey species that several known studies were missing (since lagomorph terms did not appear in bibliographic records contained in the databases). We used the same keywords for each predator (“scientific binomial name of the predator” AND diet), including all publications up to August 1st 2015. We then examined each article to determine whether lagomorphs were reported as prey items in the diet of these predators and used those studies to perform our analysis (for more details on the search see Table S1).

Further, we filtered our results to select dietary studies that comprised several preys, discarding those incomplete cases where consumption of one or few items were reported, as they are not representative of the whole diet. Moreover we selected studies where the total number of prey items was informed, discarding those where information was incomplete or insufficient to accurately extract a proportion of prey items consumed over the total number of prey items. Within each study found we compiled the following information to perform the meta-analysis: (1) Title, authors and year; (2) predator species (identifying the class, family and if they were native or introduced); (3) lagomorph species consumed (either *O. cuniculus* or *L. europaeus*); (4) continent where the study was carried out; (5) frequency of occurrence of a prey item (for each lagomorph and for subsets of native preys), and (6) number of total prey items in the study. We used the number of prey items consumed (“consumption frequency,” Berlow et al., 2004) that is confident measure to estimate the interaction strength between these two trophic levels. We did not include biomass consumed because studies reporting this information are scarce. Moreover, further calculation of prey biomass is methodologically impractical because of the lack of information needed to do it (e.g., knowing the biomass of every species reported as prey item, actual mass of individuals recognized as dietary items, etc.). However, we calculate the ratio between the lagomorphs mass and the predators mass as a descriptive way to know if these preys represent an important input of energy in relation to the predator body size. For this, we used the average adult weight of the lagomorphs and divide it by the adult average weight of each predator, using in each case, the lagomorph consumed, either *O. cuniculus*, *L. europaeus* or both separately (if they predator consumed both).

### Data analysis

We used meta-analytical tools to extract the data from published studies on the interaction links between predators and preys. Meta-analysis allows to aggregation and quantification each study result (individual “effect size”) to a data set in a way that make them statistically comparable in a common measure (Glass, 1976), where the sample size (*n*) is responsible for its accuracy (weight). Meta-analysis needs to fulfill several conditions which are critical to a sound analysis (e.g., they depend on the number, heterogeneity and quality of researches included; Viechtbauer, 2010; Higgins & Thompson, 2002). In our study we were able to meet them since we obtained a large amount of data hence making this analysis reliable. We used the raw proportion of a given event (in this study, a particular predator–prey link) over the total amount of events (all links) to obtain a common effect size. In particular we performed four separate meta-analyses, all based on the proportion of a given prey item present on the predators’ diet. We interpreted the presence of a prey item in the diet as the existence of an interaction link (consumption), and its proportion as the strength of this link.

The four meta-analyses were focused on measuring the common effect size (meaning the average consumption link with a particular prey) calculating the proportion over the total prey items of: (1) lagomorphs, (2) most consumed native preys in the presence of lagomorphs (3) the most consumed native preys in absence of lagomorphs and (4) a random subset of native preys, also in presence of lagomorphs. For the first analysis, we used all studies where lagomorphs were present as prey items measuring their proportion in the diet. For the second, we used these same studies (lagomorphs present) choosing the most consumed native prey item for each predator species within the study. For the third, we used the remaining studies (where lagomorphs where not found as prey items) choosing the most consumed native prey item for each predator species within the study. For the last analysis, we randomly selected 50 studies (lagomorphs present) and in each one of them we randomly picked one native prey-predator interaction.

The analyses were performed using the ‘metafor’ package v.1.9-7 on R software (Viechtbauer, 2010; R Development Core Team, 2012). We used the ‘escalc’ function to calculate the effect size with the measure ‘PR’: PR =*x*_*i*_∕*n*_*i*_ which corresponds to the raw proportion that a given event is expected to happen (*x*_*i*_), over the total number of events (*n*_*i*_) (Viechtbauer, 2010). We conducted our meta-analysis with a random-effects model approach, since the effects size may vary among type of studies and species (Viechtbauer, 2010). To evaluate the source of heterogeneity in our meta-analyses we used the *I*^2^ statistic which estimates (in percent) how much of the total variability in the effect size estimate is composed by heterogeneity between studies and how much due to sampling variability (within studies variability). Low *I*^2^ values (<30%) are the result of heterogeneity due sample error, while higher values (>75%) are the result of heterogeneity due differences between studies (Higgins & Thompson, 2002). We also used the additional information of each study described above (native or exotic predator species, family, etc.) as moderator variables. These categorical variables can be used in a meta-analysis to separate and compare the effect sizes of two or more different groups in a similar way than in an ANOVA (Van Houwelingen, Arends & Stijnen, 2002; Viechtbauer, 2010). This allowed us to assess differences in the effects size (interaction strength) related to the location, and the predators or prey identity.

## Results

### Bibliographic search

We found a total of 156 studies on the diet of predators that could potentially consume lagomorphs in their exotic range, from which 131 (84% of the total) reported either *O. cuniculus*, *L. europaeus* or both as prey items. Many of those studies evaluated more than one species of predator, and therefore the total number of interactions with lagomorphs reached 210. For the same reason, from the remaining 25 studies where lagomorphs did not appear in the diet we were able to extract 30 different predator-native prey interactions. In total 43 species (17 mammals and 26 birds), from nine different families (Accipitridae, Canidae, Cathartidae, Dasyuridae, Falconidae, Felidae, Mustelidae, Strigidae, Tytonidae), feed upon the two species of lagomorphs. From these, two large eagles (*Aquila audax*, *n* = 23 and *Geranoaetus melanoleucus*, *n* = 11) and a big owl (*Bubo magellanicus*, *n* = 11), two foxes (*Vulpes vulpes*, *n* = 18 and *Lycalopex culpaeus*, *n* = 16), a large felid (*Puma concolor*, *n* = 13) and a large canid (*Canis lupus dingo*, *n* = 9), account for 101 of the 210 links measured with Lagomorphs. The geographical regions were fairly well represented in published articles, being South America (*n* = 86) slightly more represented than Oceania (*n* = 70) (see detailed results in Table S1). The studies on these two regions were mainly conducted in environments where open areas as steppe or savanna-like physiognomy are predominant, with a few exceptions.

### Meta-analysis

**Figure 1:**
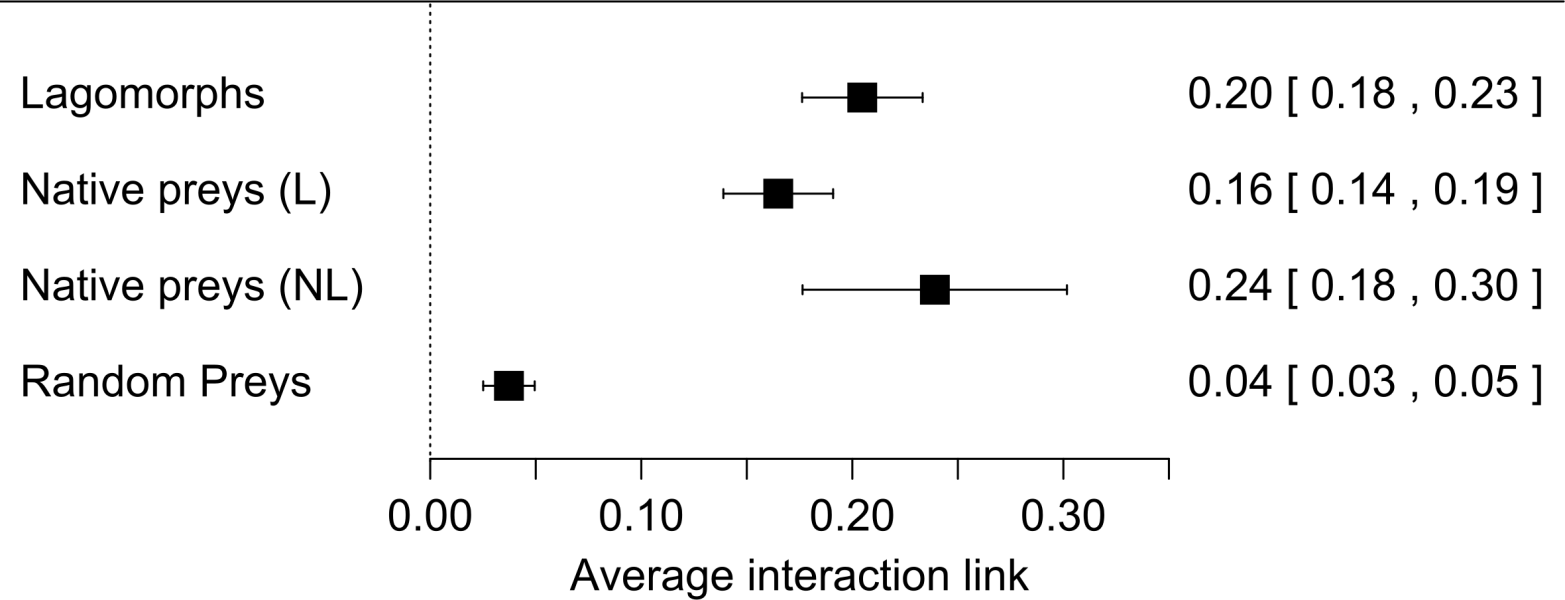
Forest plot for the four meta-analyses performed. Each black square represents the effect size (±95% CI), interpreted as the interaction strength. Numbers are the effect size for each analysis (between brackets: ±95% CI). (A) Lagomorphs: analysis of the interaction between exotic lagomorphs and predators; (B) Native preys (L): results of the most consumed native prey in presence of lagomorphs in the diet; (C) Native prey (NL): results of the most consumed native prey in absence of lagomorphs; (D) Random preys: results of randomly selected native prey-consumers interactions in presence of lagomorphs in their diet.

Our first meta-analysis consisting in the proportion of lagomorphs as prey items (with 210 measured interactions) gave us an effect size (±se) of 0.20 (±0.02) (Fig. 1, see details in Fig. S1). The total heterogeneity over the total variability (*I*^2^ = 99.98%) shows that the variability in effect estimates is due the heterogeneity between studies rather than error sampling within each study. This is expected in all our analyses since they cover several predator species and there is no reason to expect a common value. The second meta-analysis, where we calculated the proportion of the most consumed native preys when lagomorphs were present (152 interaction links), resulted in a lower effect size of 0.17 (se ± 0.01) (*I*^2^ = 99.87%) (Fig. 1, see details Fig. S2). When we analyzed the proportion of the most consumed native prey when lagomorphs were not present (30 interaction links), we obtained a higher effect size of 0.24 (se ± 0.03) (*I*^2^ = 99.22%) (Fig. 1, see details Fig. S3 ). Finally, when we analyzed the 50 interaction links randomly selected between native preys-predators we found an effect size much lower of 0.04 (se ± 0.01) (*I*^2^ = 99.70%) (Fig. 1, see details Fig. S4).

**Figure 2:**
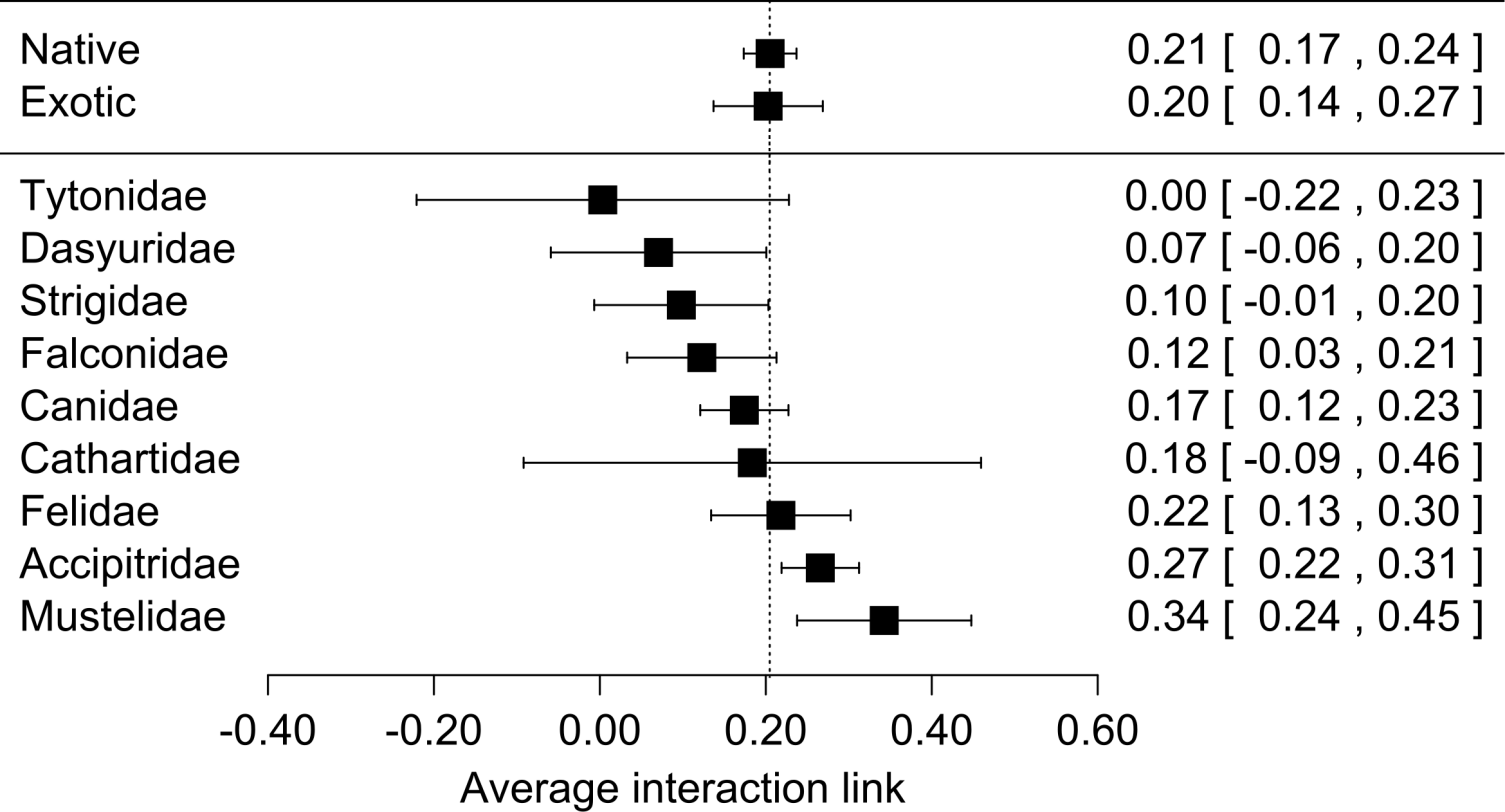
Forest plot for the meta-analyses performed for lagomorphs as preys using two moderator variables: if predators are native or exotics (Origin) and the Family of each predator species (predator family). Each black square represents the effect size (±95% CI) corresponding to the interaction strength. Numbers correspond to the effect size for each category in the moderator variables (between brackets: ±95% CI).

When we used predator origin as a moderator variable in our analysis we found that both native and exotic predator species preyed on lagomorphs equally (Fig. 2, Table S2). In total we found that 18 of 43 predator species yielded significant results in their effect sizes (Table S2). These species come from five families which presented significant results in their effect sizes as groups (Accipitridae, Falconidae, Canidae, Felidae and Mustelidae, Fig. 2, Table S2). Finally we did not find significant differences between the consumption of the two lagomorph species nor between the regions studied. However they were slightly more consumed in Oceania than South-Amercia; and *O. cuniculus* formed slightly stronger links than *L. europaeus* (Table S2).

**Figure 3:**
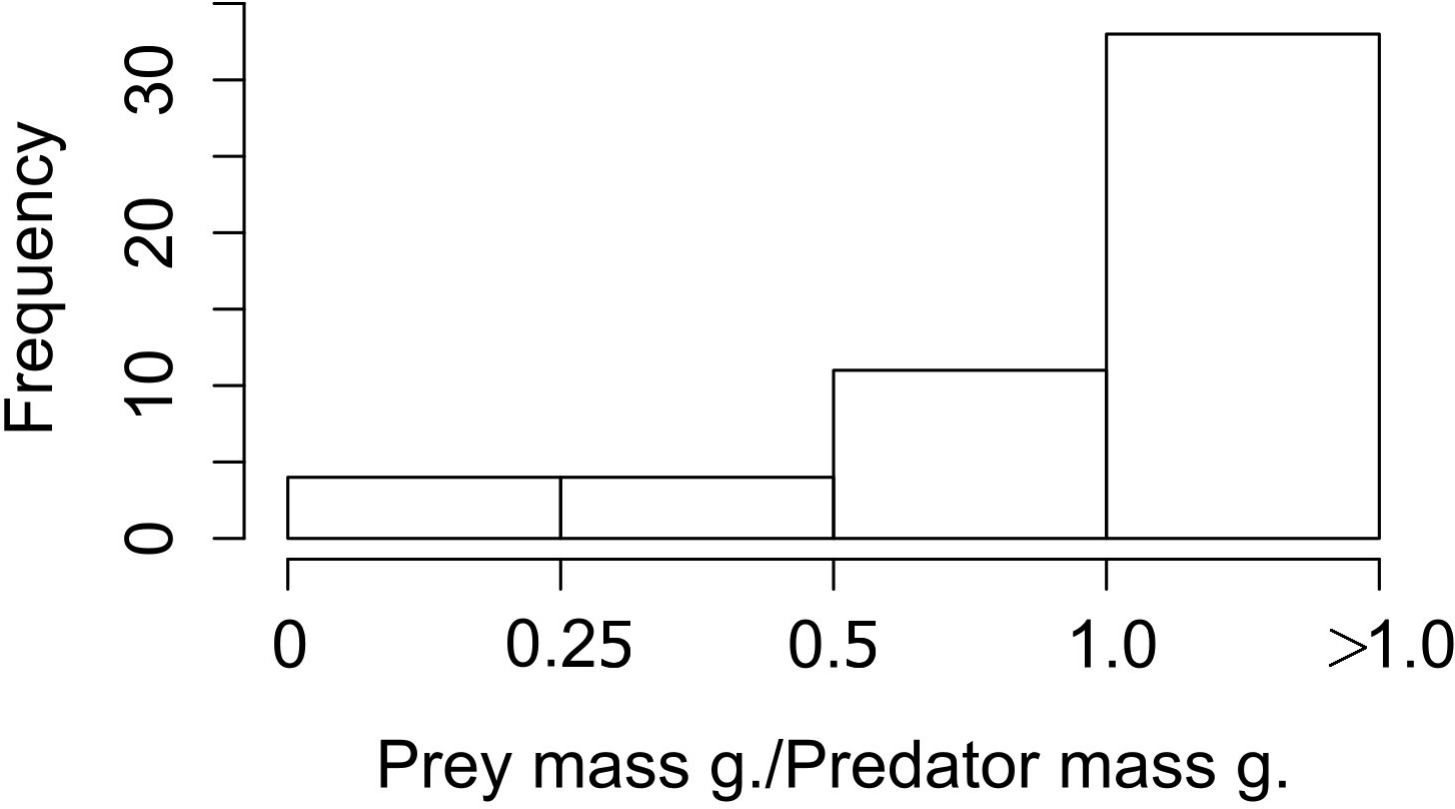
Frequency of ratios between Lagomorph weights and Predator weigths. Number of cases (frequency) that the ratio between the lagomorphs weight and the predator weights less than 0.25, between 0.25 and 0.5, 0.5–1 or more than 1.

When we looked at the relationship between the lagomorphs weight and their predators weights (52 interactions in total), we found that the ratio prey mass/predator mass was higher than 1 in 33 of the cases (63%; i.e., the prey as big as the predator or bigger), 11 ranged between 0.5 and 1 (21%); and from the rest, four (8%) are between 0.25 and 0.5 and four (8%) are smaller than 0.25 (Fig. 3).

## Discussion

Our results show that exotic medium sized herbivores, such as lagomorphs, entering into the food webs, providing a novel resource for both native and exotic predators, and producing strong interaction links with the predators. In fact, we found that the average exotic lagomorph- predator link was one fifth (20%) of the total prey items consumed, indicating a strong interaction, given that the average for the strongest link with native prey (when lagomorphs are not included in the diet) is about one-quarter (24%). Moreover, when lagomorphs are present, this strongest link decreases to less than 17%, while the others appear to be much lower, representing only 4%. This means that invasive lagomorphs are not only invading large areas and in huge numbers (Grigera & Rapoport, 1983; Thompson & King, 1994), and produce important top down effects on the local vegetation (Lees & Bell, 2008), but they are also producing bottom up effects, which are structuring and modifying the food webs. These modifications are also evident in the fact that a native-prey link with predators rarely exceeded the 50% of the diet (14 out of 182 cases: 7.7%). On the contrary when lagomorphs were present this exotic herbivore link with predators was less rare (29 out of 210 cases: 13.8%) and reached extreme values (>90%; Figs. S1–S4, Table S1). These strong links also occur in Lagomorphs native range with their native predators (i.e., Iberian Lynx-Rabbits; Ferrer & Negro, 2004), but in general, they are not as frequent, meaning that these new links we found deviate from those that would naturally occur in those un-invaded food webs.

It is worth to mention that our measure for interaction link might be biased by the fact that we only used prey items consumed but not the biomass of the prey. However, while biomass could give a good and complementary measure of the energy contribution from one trophic level to the next, it has some methodological limitations. For instance, the need to know the metabolic rates of the predator, digestibility of the materials ingested and actual mass of the individuals recognized in scats, pellets or guts (Klare, Kamler & Macdonald, 2011). Therefore our measure based on prey items proportion consumed is a confident measure to estimate the interaction strength between these two trophic levels. For large predator species the contribution represented by the biomass would be weaker (e.g., *Puma concolor*, Elbroch & Wittmer, 2013), but for medium and small size predator the lagomorphs biomass consumed would represent a much higher energetic input (e.g., *Geranoaetus melanoleucus* in Travaini et al., 1998, *Mustela furo* in Smith et al., 1995). Moreover those later families are the ones that have strongest links with lagomorphs (Fig. 2). Moreover, this result is also supported by the prey mass/predator mass ratio since for the majority 84% of the predators lagomorphs represent at least half of their own body mass, and in 63% lagomorphs are of the same mass or higher. This is even a higher ratio than the one found to be optimal for the prey of medium-sized predator mammals (around 45% of the predator mass, Carbone et al., 1999). This highlights that each lagomorph registered as prey item in the studies used for our meta-analysis represented a big energy input for the predators. Our general results are surely conservative since for most of the predator species analyzed here the consumption of each lagomorph as prey item is at least underestimating their contribution in terms of biomass consumed. Moreover, our results show that both large and small predators can have similar values regarding the interaction strength (see Table S2 for detailed information on each species) and differences between species could be related to other factors as prey availability or predator behavior rather than the measure used to perform our analyses.

Many of the predators we studied have plastic feeding behaviors that tend to adapt to the availability of resources (Jaksić, 1989; Marti, Korpimäki & Jaksić, 1993; Dupuy, Giraudoux & Delattre, 2009), and shift their diet weakening other links as a consequence of the establishment of a strong link with the exotic species. This fact along with lagomorph size, may exaggerate their structuring effects produced in the invaded communities. Medium sized herbivores play an important role structuring the communities either via top-down or bottom-up effects, changes in diversity and engineering ecosystems (Olff & Ritchie, 1998; Hopcraft, Olff & Sinclair, 2010). Lagomorphs may play these roles in their native range, shaping landscapes and maintaining top carnivore populations (Delibes & Hiraldo, 1981; Lees & Bell, 2008), demonstrating their importance to the whole ecosystem. However, this role is the result of their co-evolution in their native communities, and is expected to be balanced over time. Our results show that changes in the food web structure are currently produced at a global scale in the novel predators-lagomorphs food webs, potentially altering the whole community.

Interestingly native and exotic predators responded similarly preying on lagomorphs with the same strength (Fig. 2). This shows the importance of these prey for carnivores regardless whether they co-evolved with them or not and highlight their feeding plasticity. The strong interaction between predators and exotic lagomorphs has huge impact on the invaded communities. First, as indicated in this study, lagomorphs represent a new and generally abundant food subsidy to the consumers (Grigera & Rapoport, 1983; Lees & Bell, 2008). This can have a positive effect on predators, where their survival, reproduction and total abundances are expected to increase with the new availability of resources (Tablado et al., 2010). However, this may have negative effects on the native prey populations, leading to a case of apparent competition (hyperpredation), where a prey species adapted to high predation may have a competitive advantage and can cause the crash of a native prey populations (Courchamp, Langlais & Sugihara, 2000), or where a shift in the diet of an increased population of predators does not necessarily means a decrease in the numbers of individuals hunted (Holt, 1977). Such food webs relying on one abundant resource are vulnerable to cascade effects when perturbed and have their stability and maintenance may be compromised (O’Gorman & Emmerson, 2009).

These novel and imbalanced food webs, where there is a very strong link between two trophic levels that do not occur naturally (Kokkoris, Troumbis & Lawton, 1999; Kokkoris et al., 2002), may have several implications in conservation, as they are vulnerable to sudden changes (McCann, Hastings & Huxel, 1998; Bascompte, Melián & Sala, 2005; Rooney et al., 2006). This is of major concern considering that lagomorphs are game species and therefore susceptible to overharvesting by humans. They also have experienced large population decreases in their native range due to outbreaks of disease that endanger their local populations and also the persistence of the top predator populations that depend on them (Ferrer & Negro, 2004; Delibes-Mateos, Ferreras & Villafuerte, 2007; Lees & Bell, 2008; Moleón et al., 2009). This scenario of a prey strongly interacting with its predators in a food web may lead invaded systems to an unstable equilibrium, with any disruption having potentially catastrophic results for its diversity (O’Gorman & Emmerson, 2009).

Medium-sized herbivores that invade an ecosystem in large numbers have the potential to deeply modify the abundances of individuals of the next trophic level, and the interactions among native species and even with other exotic ones. Here we show that exotic lagomorphs can be replacing the native ones in their role as food source, leading them to an ecological extinction (Novaro, Funes & Susan Walker, 2000; Lambertucci et al., 2009). Literature on invasive species management, lagomorphs in particular, is abundant but it is mainly focused on the potential loss of biodiversity and the economic cost of their presence (Veitch & Clout, 2002; Schlaepfer, Sax & Olden, 2011). Our results and other works highlight the importance to properly manage populations of exotic herbivore prey species taking into account ecological processes as competition with other herbivores and the possible influence of hyperpredation (Cooke, Jones & Gong, 2011; Bird et al., 2012; Cooke, 2012; Wittmer et al., 2013). Even theoretical work suggest the need of a slow removal of these primary preys to avoid sudden changes in predation rates on the secondary preys leading to their extinction (Serrouya et al., 2015). This is of a great concern as we showed that exotic lagomorphs have the potential to or has already become the primary prey for many consumers. The eradication or any control strategy that affect their abundances should be carried out with caution since they will surely have a great impact on both native and exotic predators, native prey populations and ultimately on the entire community. Future studies on the matter should analyze the diversity of native prey, and the original interaction strength with native predators in the absence of lagomorphs in order to understand the strong effect that the latter produce in the invaded areas. Additionally, studies of lagomorph’s effect on the fitness of predators that consume them are important to provide a comprehensive understanding of the ecological processes related to the invasion.

## Supplemental Information

10.7717/peerj.2273/supp-1 Figure S1 Forest plot Lagomorphs as preys Forest plot for the meta-analysis on the interaction strength between introduced lagomorphs as preys and the native predators. Each black square represents an interaction link between lagomorphs and their predators (*n* = 210), bars are the CI (±95% CI) for each proportion. Dashed line is the mean interaction for lagomorphs. The black diamond represents the mean proportion for this analysis (±95% CI).

10.7717/peerj.2273/supp-2 Figure S2 Forest plot Native Preys W Lagomorphs Forest plot for the meta-analysis on the interaction strength between the most consumed native preys and the native predator in presence of lagomorphs in their diet. Each black square represents an interaction link between most consumed native prey and their predators (*n* = 152), bars are the confidence interval (±95% CI) for each proportion. Dashed line is the mean interaction for lagomorphs. The black diamond represents the mean proportion for this analysis (±95% CI).

10.7717/peerj.2273/supp-3 Figure S3 Forest plot Native Preys W/o Lagomorphs Forest plot for the meta-analysis on the interaction strength between the most consumed native preys and the native predator in absence of lagomorphs in their diet.. Each black square represents an interaction link between most consumed native prey and their predators (*n* = 30), bars are the confidence interval (±95% CI) for each proportion. Dashed line is the mean interaction for lagomorphs. The black diamond represents the mean proportion for this analysis (±95% CI).

10.7717/peerj.2273/supp-4 Figure S4 Forest plot Random Prey Forest plot for the meta-analysis on the interaction strength between random native preys and the native predator in presence of lagomorphs in their diet. Each black square represents an interaction link between random native prey and their predators (*n* = 50), bars are the confidence interval (±95% CI) for each proportion. Dashed line is the mean interaction for lagomorphs. The black diamond represents the mean proportion for this analysis (±95% CI).

10.7717/peerj.2273/supp-5 Figure S5 PRISMA Flow Diagram PRISMA Flow Diagram for the meta-analysis performed in this study.

10.7717/peerj.2273/supp-6 Table S1 List of all interactions links extracted from the bibliography List of all interactions links extracted from the bibliography used to perform the four meta-analysis. Columns describe the authors and year of each study, xi=number of prey item in each category, ni= number of total prey items in each study, the predator identities, families and classes, the region where the study was performed (either South America or Oceania), the origin of the species in that region (either native or exotic), and the group signaling in which meta-analysis was used. The complete bibliography set for these studies is provided cited below. Note: repeated predator species within the same study correspond to the presence of both lagomorphs (*Lepus europaeus* and *Oryctolagus cuniculus*) that were analyzed as two separate links, we use “*” to denote the links that corresponds to *O. cuniculus (and not L. europaeus)*.

10.7717/peerj.2273/supp-7 Table S2 Detailed results of the four meta-analyses performed in this study items. Detailed results of the four meta-analyses performed in this study. Estimate is the effect size interpreted as interaction strength. The first four results correspond to the four separated meta-analysis: lagomorphs as prey, most consumed preys in presence of lagomorphs, random preys in presence of lagomorphs and the most consumed preys in absence of lagomorphs. The results for each moderator variables correspond to the analysis of lagomorphs as exotic preys in the diet of the predators. Signif. codes *p*-value: ‘^∗∗∗^′ = 0 > *p* > 0.001, ‘^∗∗^′ = 0.001 > *p* > 0.01, ‘^∗^′ = 0.01 > *p* > 0.05, ‘.′ = 0.05 > *p* > 0.1, ‘′ = *p* > 0.1.

10.7717/peerj.2273/supp-8 Table S3 PRISMA Check list PRISMA Check list for this study

## Additional Information and Declarations

### Data Availability

The following information was supplied regarding data availability: The raw data has been supplied as a Supplemental Dataset.
