# Supplementary figures and images for: Medium-sized exotic prey create novel food webs: the case of predators and scavengers consuming lagomorphs
Source: PeerJ. 2016 Jul 27;4:e2273. doi: 10.7717/peerj.2273 (PMC4974932; doi:10.7717/peerj.2273)

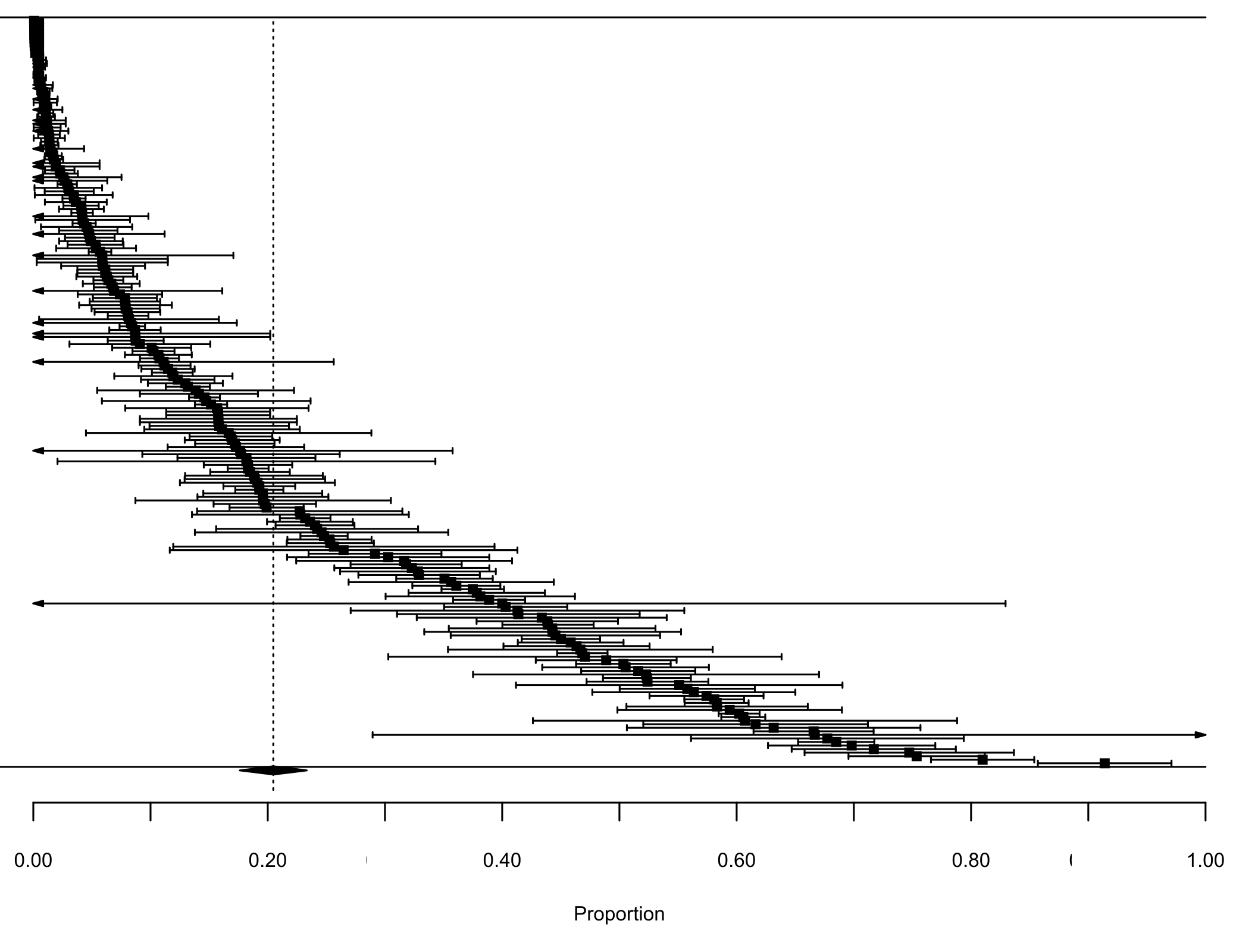

Supplement: Figure S1 — Forest plot for the meta-analysis on the interaction strength between introduced lagomorphs as preys and the native predators. Each black square represents an interaction link between lagomorphs and their predators (n = 210), bars are the CI (±95% CI) for each proportion. Dashed line is the mean interaction for lagomorphs. The black diamond represents the mean proportion for this analysis (±95% CI). [file peerj-04-2273-s001.png]

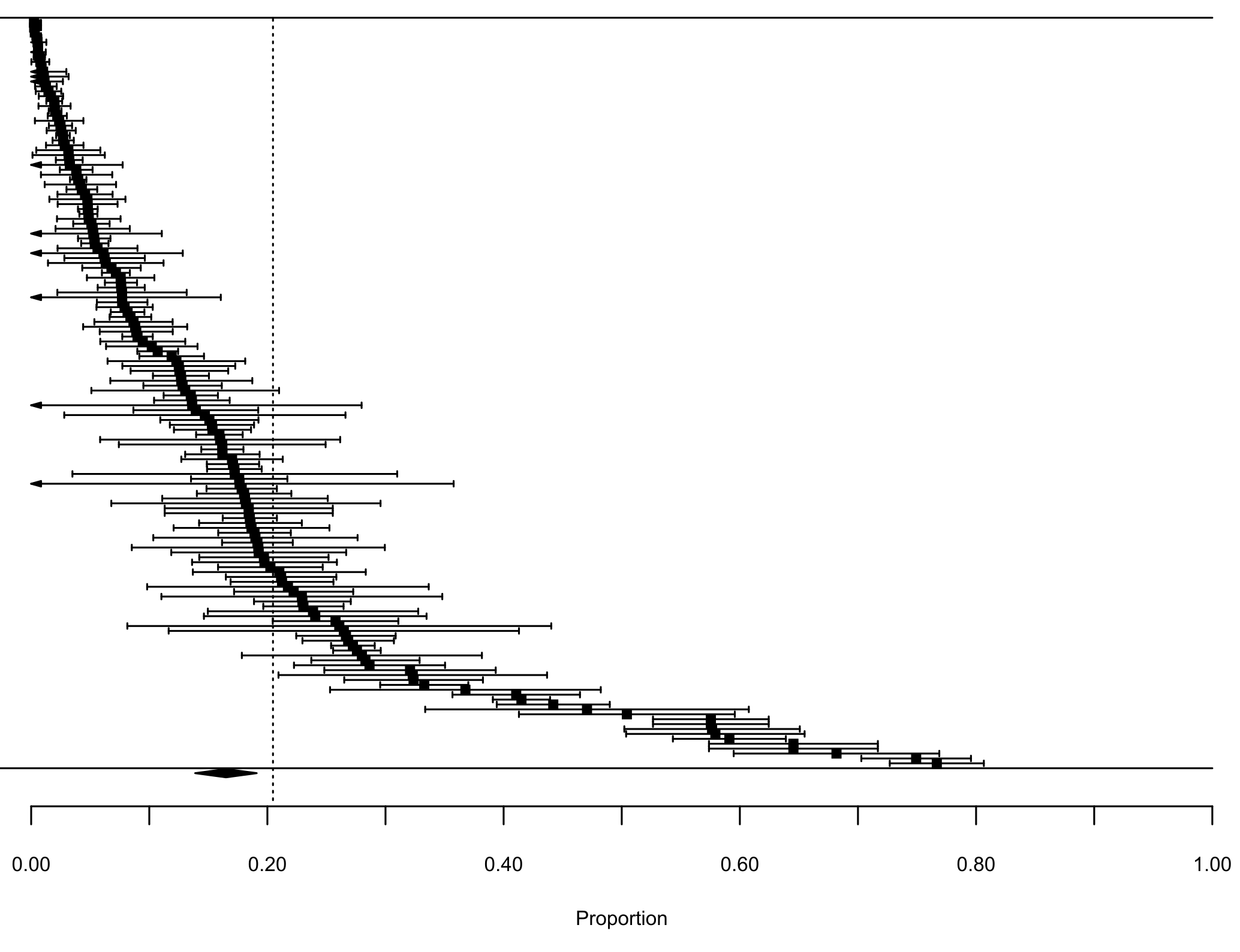

Supplement: Figure S2 — Forest plot for the meta-analysis on the interaction strength between the most consumed native preys and the native predator in presence of lagomorphs in their diet. Each black square represents an interaction link between most consumed native prey and their predators (n = 152), bars are the confidence interval (±95% CI) for each proportion. Dashed line is the mean interaction for lagomorphs. The black diamond represents the mean proportion for this analysis (±95% CI). [file peerj-04-2273-s002.png]

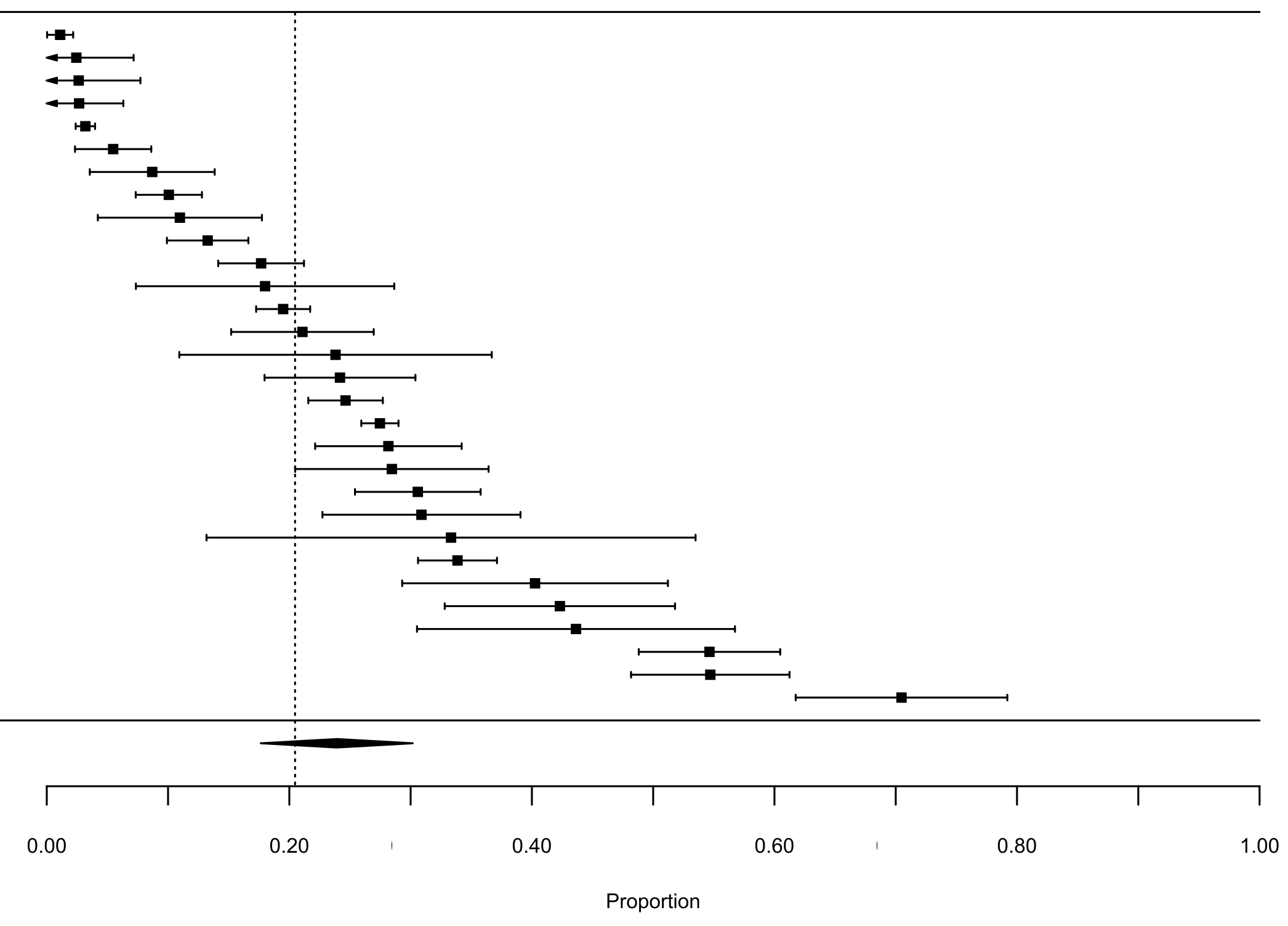

Supplement: Figure S3 — Forest plot for the meta-analysis on the interaction strength between the most consumed native preys and the native predator in absence of lagomorphs in their diet.. Each black square represents an interaction link between most consumed native prey and their predators (n = 30), bars are the confidence interval (±95% CI) for each proportion. Dashed line is the mean interaction for lagomorphs. The black diamond represents the mean proportion for this analysis (±95% CI). [file peerj-04-2273-s003.png]

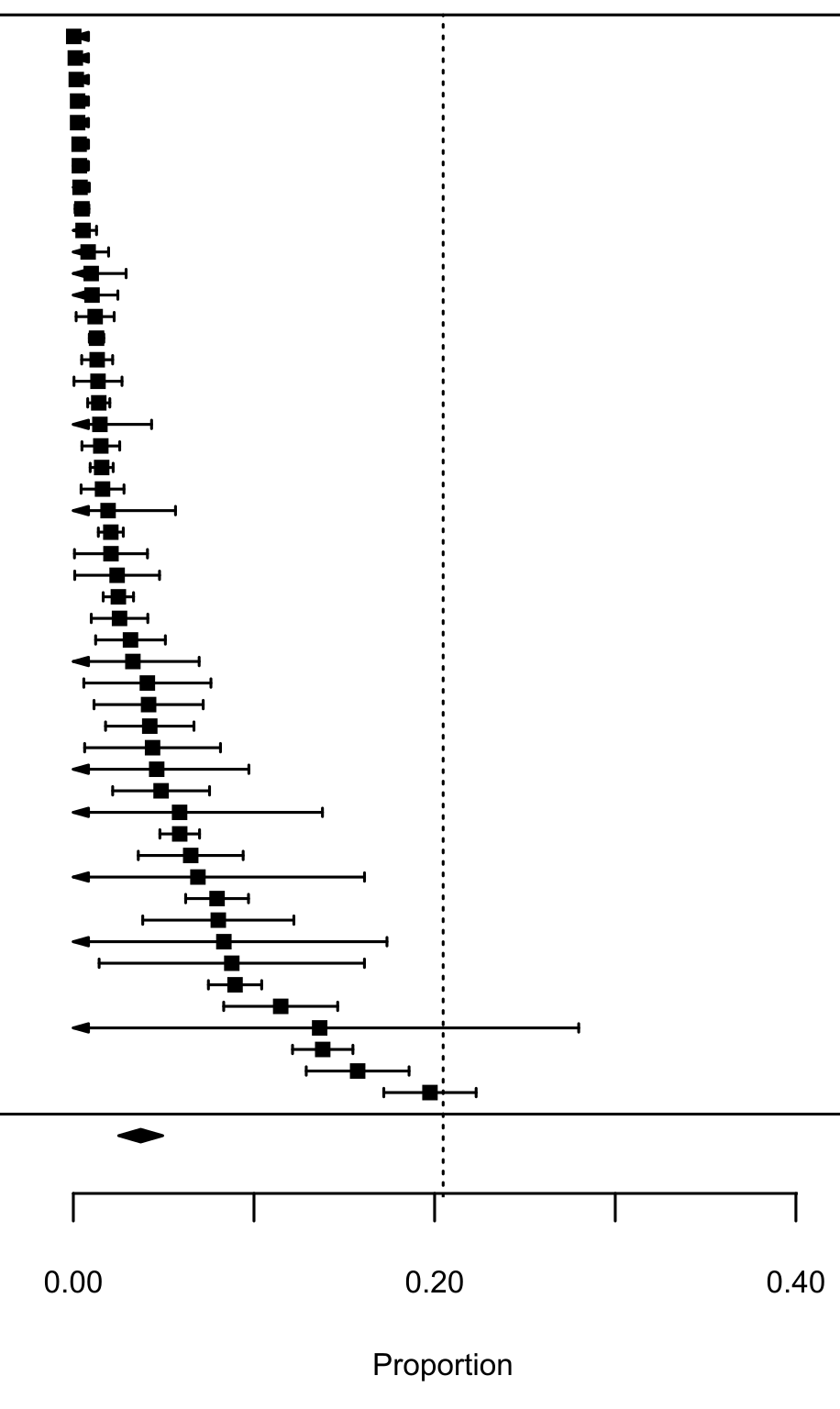

Supplement: Figure S4 — Forest plot for the meta-analysis on the interaction strength between random native preys and the native predator in presence of lagomorphs in their diet. Each black square represents an interaction link between random native prey and their predators (n = 50), bars are the confidence interval (±95% CI) for each proportion. Dashed line is the mean interaction for lagomorphs. The black diamond represents the mean proportion for this analysis (±95% CI). [file peerj-04-2273-s004.png]

**Fig. Prisma Flow Diagrama to Include When Reporting Meta-analysis**

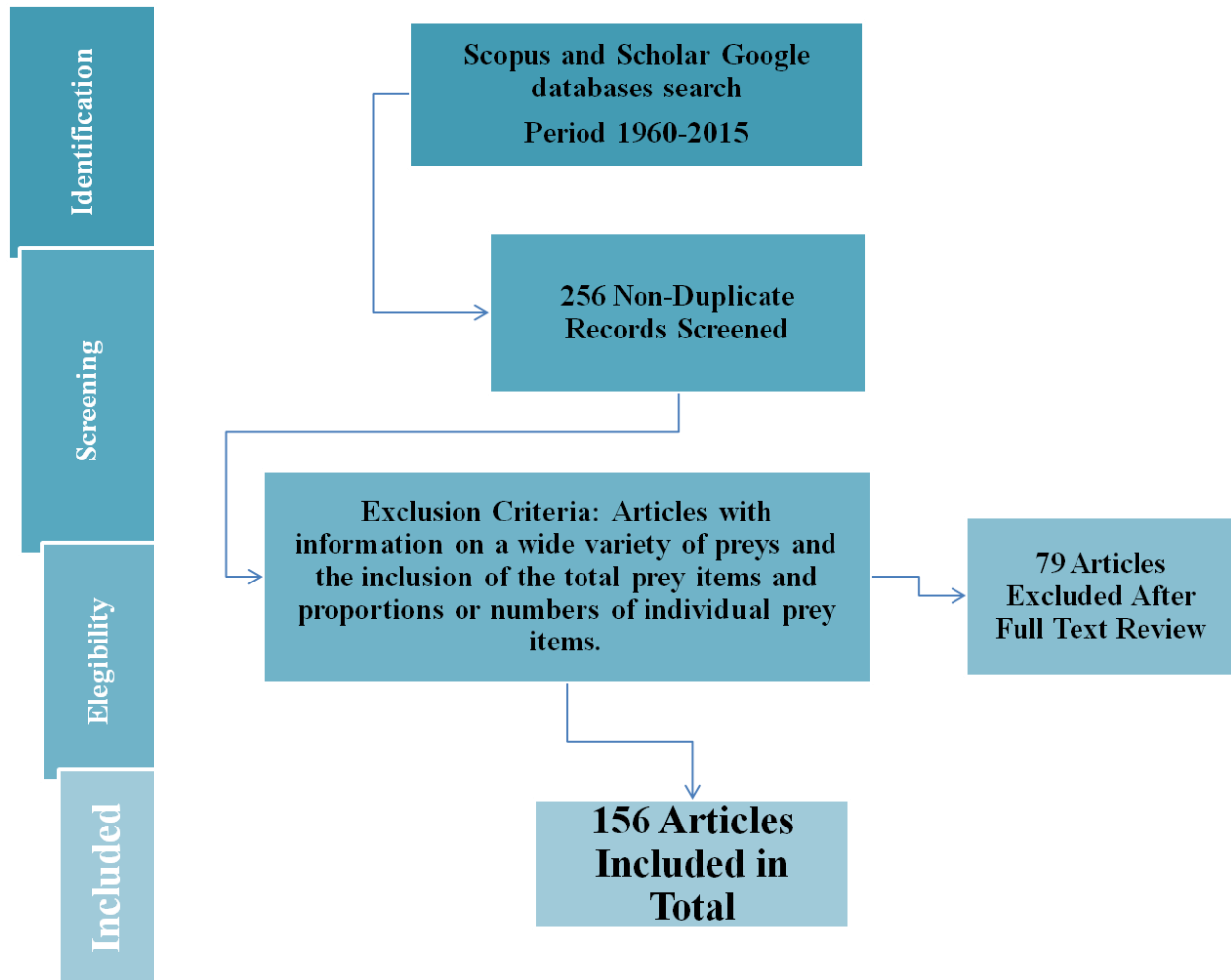

Supplement: Figure S5 — PRISMA Flow Diagram for the meta-analysis performed in this study. [file peerj-04-2273-s005.pdf]
