# Supplementary material for: Medium-sized exotic prey create novel food webs: the case of predators and scavengers consuming lagomorphs
Source: PeerJ. 2016 Jul 27;4:e2273. doi: 10.7717/peerj.2273 (PMC4974932; doi:10.7717/peerj.2273)
Supplement: Table S1 — List of all interactions links extracted from the bibliography used to perform the four meta-analysis. Columns describe the authors and year of each study, xi=number of prey item in each category, ni= number of total prey items in each study, the predator identities, families and classes, the region where the study was performed (either South America or Oceania), the origin of the species in that region (either native or exotic), and the group signaling in which meta-analysis was used. The complete bibliography set for these studies is provided cited below. Note: repeated predator species within the same study correspond to the presence of both lagomorphs (Lepus europaeus and Oryctolagus cuniculus) that were analyzed as two separate links, we use “*” to denote the links that corresponds to O. cuniculus (and not L. europaeus). [file peerj-04-2273-s006.pdf]

Supporting information for

Medium-sized exotic prey create novel food webs: the case of predators and scavengers consuming lagomorphs

Authors

Facundo Barbar[1], Fernando Hiraldo[2], Sergio A. Lambertucci[1].

Authors' affiliations

[1] Grupo de Biología de la Conservación, Laboratorio Ecotono INIBIOMA (CONICET- Universidad Nacional del Comahue).

[2] Departamento de Biología de la Conservación, Estación Biológica Doñana (CSIC) Sevilla, España.

Table S1. List of all interactions links extracted from the bibliography used to perform the four meta-analysis. Columns describe the authors and year of each study, xi=number of prey item in each category, ni= number of total prey items in each study, the predator identities, families and classes, the region where the study was performed (either South America or Oceania), the origin of the species in that region (either native or exotic), and the group signaling in which meta-analysis was used. The complete bibliography set for these studies is provided cited below. Note: repeated predator species within the same study correspond to the presence of both lagomorphs (*Lepus europaeus* and *Oryctolagus cuniculus*) that were analyzed as two separate links, we use "*"to denote the links that corresponds to *O. cuniculus (and not L. europaeus).*

Study	xi	ni	Predator	Predator Family	Predator class	Predator origin	Region	Group
Allen et al. 2012	13	1460	*Canis lupus dingo*	Canidae	Mammals	Exotic	Oceania	Lagomorphs
Alterio & Moller. 1997	35	75	*Mustela erminea*	Mustelidae	Mammals	Exotic	Oceania	Lagomorphs
Alterio & Moller. 1997	42	62	*Mustela furo*	Mustelidae	Mammals	Exotic	Oceania	Lagomorphs
Arriagada et al. 2011	54	122	*Geranoaetus melanoleucus*	Accipitridae	Birds	Native	Southamerica	Lagomorphs
Astorga Saavedra 2013	2	42	*Lycalopex culpaeus*	Canidae	Mammals	Native	Southamerica	Lagomorphs
Aumann 1988	438	1766	*Accipiter fasciatus*	Accipitridae	Birds	Native	Oceania	Lagomorphs
Aumann 2011	1	272	*Accipiter fasciatus*	Accipitridae	Birds	Native	Oceania	Lagomorphs
Aumann 2011	53	119	*Aquila audax*	Accipitridae	Birds	Native	Oceania	Lagomorphs
Aumann 2011	8	734	*Falco berigora*	Falconidae	Birds	Native	Oceania	Lagomorphs
Aumann 2011	76	1826	*Hamirostra melanosternon*	Accipitridae	Birds	Native	Oceania	Lagomorphs
Aumann 2011	8	597	*Hieraaetus morphnoides*	Accipitridae	Birds	Native	Oceania	Lagomorphs
Baker-Gabb 1981	82	477	*Circus approximans*	Accipitridae	Birds	Native	Oceania	Lagomorphs
Baker-Gabb 1981	2	477	*Circus approximans*	Accipitridae	Birds	Native	Oceania	Lagomorphs
Baker-Gabb 1983	71	126	*Accipiter fasciatus*	Accipitridae	Birds	Native	Oceania	Lagomorphs
Baker-Gabb 1983	68	91	*Aquila audax*	Accipitridae	Birds	Native	Oceania	Lagomorphs
Baker-Gabb 1983	186	355	*Circus assimilis*	Accipitridae	Birds	Native	Oceania	Lagomorphs
Baker-Gabb 1983	114	246	*Falco berigora*	Falconidae	Birds	Native	Oceania	Lagomorphs
Baker-Gabb 1983	1	177	*Falco cenchroides*	Falconidae	Birds	Native	Oceania	Lagomorphs
Baker-Gabb 1983	135	335	*Falco subniger*	Falconidae	Birds	Native	Oceania	Lagomorphs
Baker-Gabb 1983	111	159	*Haliastur sphenurus*	Accipitridae	Birds	Native	Oceania	Lagomorphs
Baker-Gabb 1983	159	211	*Hamirostra melanosternon*	Accipitridae	Birds	Native	Oceania	Lagomorphs
Baker-Gabb 1983	114	159	*Hieraaetus morphnoides*	Accipitridae	Birds	Native	Oceania	Lagomorphs
Baker-Gabb 1984	96	190	*Accipiter fasciatus*	Accipitridae	Birds	Native	Oceania	Lagomorphs
Baker-Gabb 1984	87	777	*Circus approximans*	Accipitridae	Birds	Native	Oceania	Lagomorphs
Baker-Gabb 1984	144	1338	*Falco berigora*	Falconidae	Birds	Native	Oceania	Lagomorphs
Baker-Gabb 1984	29	361	*Haliastur sphenurus*	Accipitridae	Birds	Native	Oceania	Lagomorphs

Ballejo y De Santis 2013	164	1244	*Coragyps atratus*	Cathartidae	Birds	Native	Southamerica	Lagomorphs
Belcher et al. 2007	17	414	*Dasyurus maculatus*	Dasyuridae	Mammals	Native	Oceania	Lagomorphs
Biondi et al. 2005	3	4553	*Milvago chimango*	Falconidae	Birds	Native	Southamerica	Lagomorphs
Birochio 2008	4	293	*Lycalopex gymnocercus*	Canidae	Mammals	Native	Southamerica	Lagomorphs
Bisceglia et al. 2008	2	441	*Leopardus geofroyii*	Felidae	Mammals	Native	Southamerica	Lagomorphs
Branch et al. 1996	21	335	*Puma concolor*	Felidae	Mammals	Native	Southamerica	Lagomorphs
Brook y Kutt. 2011	26	184	*Canis lupus dingo*	Canidae	Mammals	Exotic	Oceania	Lagomorphs
Brooker y Ridhpath 1980	1810	3006	*Aquila audax*	Accipitridae	Birds	Native	Oceania	Lagomorphs
Brunner et al. 1975	346	1888	*Vulpes vulpes*	Canidae	Mammals	Exotic	Oceania	Lagomorphs
Bustamante et al. 1997	837	1441	*Geranoaetus melanoleucus*	Accipitridae	Birds	Native	Southamerica	Lagomorphs
Castillo et al. 2011	4	6	*Lycalopex gymnocercus*	Canidae	Mammals	Native	Southamerica	Lagomorphs
Catry et al. 2008	19	46	*Buteo polyosoma*	Accipitridae	Birds	Native	Southamerica	Lagomorphs
Catry et al. 2008	27	406	*Phalcoboenus australis*	Falconidae	Birds	Native	Southamerica	Lagomorphs*
Catry et al. 2008	3	17	*Phalcoboenus australis*	Falconidae	Birds	Native	Southamerica	Lagomorphs
Cherrimann 2008	72	247	*Aquila audax*	Accipitridae	Birds	Native	Oceania	Lagomorphs
Coates & Wright 2003	33	109	*Vulpes vulpes*	Canidae	Mammals	Exotic	Oceania	Lagomorphs
Collins & Croft 2007	5	110	*Aquila audax*	Accipitridae	Birds	Native	Oceania	Lagomorphs
Coman 1973	376	967	*Vulpes vulpes*	Canidae	Mammals	Exotic	Oceania	Lagomorphs*
Coman 1973	1	967	*Vulpes vulpes*	Canidae	Mammals	Exotic	Oceania	Lagomorphs
Corbett y Newsome 1987	159	285	*Canis lupus dingo*	Canidae	Mammals	Exotic	Oceania	Lagomorphs
Correa & Roa 2005	2	23	*Lycalopex culpaeus*	Canidae	Mammals	Native	Southamerica	Lagomorphs
Correa & Roa 2005	1	17	*Lycalopex griseus*	Canidae	Mammals	Native	Southamerica	Lagomorphs
Correa & Roa 2005	2	29	*Oncifelis guigna*	Felidae	Mammals	Native	Southamerica	Lagomorphs
Dawson et al. 2007	340	1466	*Dasyurus maculatus*	Dasyuridae	Mammals	Native	Oceania	Lagomorphs
Debus 1984	13	83	*Aquila audax*	Accipitridae	Birds	Native	Oceania	Lagomorphs
Debus 1984	36	83	*Aquila audax*	Accipitridae	Birds	Native	Oceania	Lagomorphs*
Debus 1984	60	101	*Hieraaetus morphnoides*	Accipitridae	Birds	Native	Oceania	Lagomorphs
Debus et al. 2007	27	49	*Aquila audax*	Accipitridae	Birds	Native	Oceania	Lagomorphs*
Debus et al. 2007	4	49	*Aquila audax*	Accipitridae	Birds	Native	Oceania	Lagomorphs

Diuk-Wasser y Cassini 1998	53	139	*Galictis cuja*	Mustelidae	Mammals	Native	Southamerica	Lagomorphs
Donadio et al. 2010	47	538	*Puma concolor*	Felidae	Mammals	Native	Southamerica	Lagomorphs
Donazar et al. 1997	157	1324	*Bubo magellanicus*	Strigidae	Birds	Native	Southamerica	Lagomorphs*
Donazar et al. 1997	46	1324	*Bubo magellanicus*	Strigidae	Birds	Native	Southamerica	Lagomorphs
Ebensperger et al. 1991	9	34	*Galictis cuja*	Mustelidae	Mammals	Native	Southamerica	Lagomorphs
Ebensperger et al. 1991	15	61	*Lycalopex culpaeus*	Canidae	Mammals	Native	Southamerica	Lagomorphs
Ebensperger et al. 1991	4	134	*Tyto alba*	Strigidae	Birds	Native	Southamerica	Lagomorphs
Elbroch y Wittmer 2013	73	433	*Puma concolor*	Felidae	Mammals	Native	Southamerica	Lagomorphs
Ellis et al. 2010	1	52	*Falco peregrinus*	Falconidae	Birds	Native	Southamerica	Lagomorphs
Farias & Kittlein 2008	17	1638	*Lycalopex gymnocercus*	Canidae	Mammals	Native	Southamerica	Lagomorphs
Figueroa Rojas y Gonzales Acuña 2006	4	68	*Parabuteo unicinctus*	Accipitridae	Birds	Native	Southamerica	Lagomorphs
Figueroa Rojas y Gonzales Acuña 2006	1	68	*Parabuteo unicinctus*	Accipitridae	Birds	Native	Southamerica	Lagomorphs
Formoso et al. 2012	71	1637	*Bubo magellanicus*	Strigidae	Birds	Native	Southamerica	Lagomorphs
Franklin et al. 1999	209	405	*Puma concolor*	Felidae	Mammals	Native	Southamerica	Lagomorphs
Fuentes et al. 1993	17	28	*Asio flammeus*	Strigidae	Birds	Native	Southamerica	Lagomorphs
Fuentes et al. 1993	3	36	*Buteo polyosoma*	Accipitridae	Birds	Native	Southamerica	Lagomorphs
Fuentes et al. 2005	10	39	*Haliastur sphenurus*	Accipitridae	Birds	Native	Oceania	Lagomorphs*
Fuentes et al. 2005	1	39	*Haliastur sphenurus*	Accipitridae	Birds	Native	Oceania	Lagomorphs
Fuentes et al. 2007	114	260	*Aquila audax*	Accipitridae	Birds	Native	Oceania	Lagomorphs*
Fuentes et al. 2007	41	260	*Aquila audax*	Accipitridae	Birds	Native	Oceania	Lagomorphs
Fuentes et al. 2007	56	330	*Aquila audax*	Accipitridae	Birds	Native	Oceania	Lagomorphs*
Fuentes et al. 2007	26	330	*Aquila audax*	Accipitridae	Birds	Native	Oceania	Lagomorphs
Galuppo Gaete 2014	10	51	*Oncifelis guigna*	Felidae	Mammals	Native	Southamerica	Lagomorphs
Garcia & Kittlein 2005	215	469	*Lycalopex gymnocercus*	Canidae	Mammals	Native	Southamerica	Lagomorphs
Gillies 1998	6	36	*Mustela erminea*	Mustelidae	Mammals	Exotic	Oceania	Lagomorphs
Gillies 1998	2	5	*Mustela furo*	Mustelidae	Mammals	Exotic	Oceania	Lagomorphs
Gillies 1998	2	18	*Mustela nivea*	Mustelidae	Mammals	Exotic	Oceania	Lagomorphs

Glen & Dickman 2006	55	634	*Dasyurus maculatus*	Dasyuridae	Mammals	Native	Oceania	Lagomorphs
Glen & Dickman 2008	55	424	*Dasyurus maculatus*	Dasyuridae	Mammals	Native	Oceania	Lagomorphs
Glen et al. 2006	2	48	*Vulpes vulpes*	Canidae	Mammals	Exotic	Oceania	Lagomorphs
Glen et al. 2010	4	392	*Dasyurus geofroyii*	Dasyuridae	Mammals	Native	Oceania	Lagomorphs
Glen et al. 2011	4	68	*Canis lupus dingo*	Canidae	Mammals	Exotic	Oceania	Lagomorphs
Glen et al. 2011	10	168	*Dasyurus maculatus*	Dasyuridae	Mammals	Native	Oceania	Lagomorphs
Glen et al. 2011	9	168	*Dasyurus maculatus*	Dasyuridae	Mammals	Native	Oceania	Lagomorphs
Glen et al. 2011	4	95	*Vulpes vulpes*	Canidae	Mammals	Exotic	Oceania	Lagomorphs
Green & Osborne 1981	10	1159	*Vulpes vulpes*	Canidae	Mammals	Exotic	Oceania	Lagomorphs
Green & Osborne 1981	1	1159	*Vulpes vulpes*	Canidae	Mammals	Exotic	Oceania	Lagomorphs
Hiraldo et al. 1995	731	1254	*Geranoaetus melanoleucus*	Accipitridae	Birds	Native	Southamerica	Lagomorphs
Iriarte et al. 1989	118	371	*Lycalopex culpaeus*	Canidae	Mammals	Native	Southamerica	Lagomorphs
Iriarte et al. 1990	28	162	*Bubo magellanicus*	Strigidae	Birds	Native	Southamerica	Lagomorphs
Iriarte et al. 1990	1	823	*Circus cinereus*	Accipitridae	Birds	Native	Southamerica	Lagomorphs
Iriarte et al. 1990	85	93	*Geranoaetus melanoleucus*	Accipitridae	Birds	Native	Southamerica	Lagomorphs
Iriarte et al. 1991	297	590	*Puma concolor*	Felidae	Mammals	Native	Southamerica	Lagomorphs
Jaksic & Yañez 1980	18	114	*Bubo magellanicus*	Strigidae	Birds	Native	Southamerica	Lagomorphs
Jaksic et al. 1980	2	172	*Parabuteo unicinctus*	Accipitridae	Birds	Native	Southamerica	Lagomorphs
Jaksic et al. 1981	3	3038	*Athene cunicularia*	Strigidae	Birds	Native	Southamerica	Lagomorphs
Jaksic et al. 1981	18	114	*Bubo magellanicus*	Strigidae	Birds	Native	Southamerica	Lagomorphs
Jaksic et al. 1981	24	391	*Buteo polyosoma*	Accipitridae	Birds	Native	Southamerica	Lagomorphs
Jaksic et al. 1981	31	164	*Geranoaetus melanoleucus*	Accipitridae	Birds	Native	Southamerica	Lagomorphs
Jaksic et al. 1981	63	319	*Lycalopex culpaeus*	Canidae	Mammals	Native	Southamerica	Lagomorphs
Jaksic et al. 1981	2	172	*Parabuteo unicinctus*	Accipitridae	Birds	Native	Southamerica	Lagomorphs
Jaksic et al. 1986	4	339	*Bubo magellanicus*	Strigidae	Birds	Native	Southamerica	Lagomorphs
Jaksic et al. 1986	18	339	*Bubo magellanicus*	Strigidae	Birds	Native	Southamerica	Lagomorphs
Jaksic et al. 1990	4	932	*Lycalopex fulvipes*	Canidae	Mammals	Native	Southamerica	Lagomorphs
Jarman et al. 2007	11	1023	*Dasyurus maculatus*	Dasyuridae	Mammals	Native	Oceania	Lagomorphs
Jarman et al. 2007	14	1023	*Dasyurus maculatus*	Dasyuridae	Mammals	Native	Oceania	Lagomorphs

Jimenez & Jaksic 1988	1	1259	*Circus cinereus*	Accipitridae	Birds	Native	Southamerica	Lagomorphs
Jimenez & Jaksic 1989	9	65	*Geranoaetus melanoleucus*	Accipitridae	Birds	Native	Southamerica	Lagomorphs
Jimenez & Jaksic 1993	30	165	*Parabuteo unicinctus*	Accipitridae	Birds	Native	Southamerica	Lagomorphs
Johnson & Franklin 1991	228	397	*Leopardus geofroyii*	Felidae	Mammals	Native	Southamerica	Lagomorphs
Johnson & Franklin 1994	537	784	*Lycalopex culpaeus*	Canidae	Mammals	Native	Southamerica	Lagomorphs
Johnson & Franklin 1994	383	851	*Lycalopex griseus*	Canidae	Mammals	Native	Southamerica	Lagomorphs
King 1991	213	2522	*Mustela erminea*	Mustelidae	Mammals	Exotic	Oceania	Lagomorphs
Kirkwood et al. 2005	41	115	*Vulpes vulpes*	Canidae	Mammals	Exotic	Oceania	Lagomorphs
Lambertucci et al. 2009	122	517	*Vultur gryphus*	Cathartidae	Birds	Native	Southamerica	Lagomorphs
Lugton 1991	74	404	*Vulpes vulpes*	Canidae	Mammals	Exotic	Oceania	Lagomorphs
Lunney et al. 1990	122	613	*Vulpes vulpes*	Canidae	Mammals	Exotic	Oceania	Lagomorphs*
Lunney et al. 1990	1	613	*Vulpes vulpes*	Canidae	Mammals	Exotic	Oceania	Lagomorphs
Marlow et al. 2015	48	450	*Vulpes vulpes*	Canidae	Mammals	Exotic	Oceania	Lagomorphs
McDonald & Baker-Gabb 2006	123	638	*Falco berigora*	Falconidae	Birds	Native	Oceania	Lagomorphs
McDonald 2004	149	620	*Falco berigora*	Falconidae	Birds	Native	Oceania	Lagomorphs
McDonald et al. 2003	36	87	*Falco berigora*	Falconidae	Birds	Native	Oceania	Lagomorphs
McDonald et al. 2012	24	307	*Falco berigora*	Falconidae	Birds	Native	Oceania	Lagomorphs
McDonald et al. 2012	3	307	*Falco berigora*	Falconidae	Birds	Native	Oceania	Lagomorphs
McIntosh 1963	101	267	*Vulpes vulpes*	Canidae	Mammals	Exotic	Oceania	Lagomorphs
Micthell & Banks 2005	31	307	*Vulpes vulpes*	Canidae	Mammals	Exotic	Oceania	Lagomorphs
Molsher et al. 2000	87	757	*Vulpes vulpes*	Canidae	Mammals	Exotic	Oceania	Lagomorphs
Monserrat et al. 2005	9	390	*Bubo magellanicus*	Strigidae	Birds	Native	Southamerica	Lagomorphs
Monserrat et al. 2005	7	193	*Buteo polyosoma*	Accipitridae	Birds	Native	Southamerica	Lagomorphs
Monserrat et al. 2005	181	516	*Geranoaetus melanoleucus*	Accipitridae	Birds	Native	Southamerica	Lagomorphs
Monteverde & Piudo 2011	16	34	*Lycalopex culpaeus*	Canidae	Mammals	Native	Southamerica	Lagomorphs
Morley 1999	247	305	*Mustela furo*	Mustelidae	Mammals	Exotic	Oceania	Lagomorphs
Muñoz & Maura 1990	23	44	*Lycalopex culpaeus*	Canidae	Mammals	Native	Southamerica	Lagomorphs
Murphy & Dowding 1994	23	95	*Mustela erminea*	Mustelidae	Mammals	Exotic	Oceania	Lagomorphs

Murphy & Dowding 1995	23	145	*Mustela erminea*	Mustelidae	Mammals	Exotic	Oceania	Lagomorphs
Murphy et al. 2004	31	98	*Mustela erminea*	Mustelidae	Mammals	Exotic	Oceania	Lagomorphs
Murphy et al. 2004	130	266	*Mustela furo*	Mustelidae	Mammals	Exotic	Oceania	Lagomorphs
Murphy et al. 2008	12	874	*Mustela erminea*	Mustelidae	Mammals	Exotic	Oceania	Lagomorphs
Nabte et al. 2006	21	1232	*Bubo magellanicus*	Strigidae	Birds	Native	Southamerica	Lagomorphs
Newsome 1984	113	1102	*Canis lupus dingo*	Canidae	Mammals	Exotic	Oceania	Lagomorphs
Newsome et al. 2014	1	254	*Canis lupus dingo*	Canidae	Mammals	Exotic	Oceania	Lagomorphs
Novaro et al. 2000	18	79	*Leopardus geofroyii*	Felidae	Mammals	Native	Southamerica	Lagomorphs
Novaro et al. 2000	142	562	*Lycalopex culpaeus*	Canidae	Mammals	Native	Southamerica	Lagomorphs
Novaro et al. 2000	2	562	*Lycalopex culpaeus*	Canidae	Mammals	Native	Southamerica	Lagomorphs
Novaro et al. 2000	9	61	*Lycalopex gymnocercus*	Canidae	Mammals	Native	Southamerica	Lagomorphs
Novaro et al. 2000	61	99	*Puma concolor*	Felidae	Mammals	Native	Southamerica	Lagomorphs
Olsen et al. 2006 (a)	4	22	*Accipiter fasciatus*	Accipitridae	Birds	Native	Oceania	Lagomorphs
Olsen et al. 2006 (a)	1	52	*Falco peregrinus*	Falconidae	Birds	Native	Oceania	Lagomorphs
Olsen et al. 2006 (b)	19	118	*Aquila audax*	Accipitridae	Birds	Native	Oceania	Lagomorphs
Olsen et al. 2006 (b)	4	116	*Haliaeetus leucogaster*	Accipitridae	Birds	Native	Oceania	Lagomorphs
Olsen et al. 2010	274	1421	*Aquila audax*	Accipitridae	Birds	Native	Oceania	Lagomorphs*
Olsen et al. 2010	91	1421	*Aquila audax*	Accipitridae	Birds	Native	Oceania	Lagomorphs
Olsen et al. 2010	62	192	*Hieraaetus morphnoides*	Accipitridae	Birds	Native	Oceania	Lagomorphs
Olsen et al. 2010	2	192	*Hieraaetus morphnoides*	Accipitridae	Birds	Native	Oceania	Lagomorphs
Olsen et al. 2013	26	136	*Haliastur sphenurus*	Accipitridae	Birds	Native	Oceania	Lagomorphs
Olsen et al. 2014	227	629	*Aquila audax*	Accipitridae	Birds	Native	Oceania	Lagomorphs*
Olsen et al. 2014	41	260	*Aquila audax*	Accipitridae	Birds	Native	Oceania	Lagomorphs
Palacios et al. 2012	2	23	*Leopardus colocolo*	Felidae	Mammals	Native	Southamerica	Lagomorphs
Palacios et al. 2012	14	178	*Leopardus geofroyii*	Felidae	Mammals	Native	Southamerica	Lagomorphs
Palacios et al. 2012	52	422	*Lycalopex culpaeus*	Canidae	Mammals	Native	Southamerica	Lagomorphs
Palacios et al. 2012	19	394	*Lycalopex gymnocercus*	Canidae	Mammals	Native	Southamerica	Lagomorphs
Pavez et al. 1992	274	624	*Geranoaetus melanoleucus*	Accipitridae	Birds	Native	Southamerica	Lagomorphs
Pessino et al. 2001	12	244	*Puma concolor*	Felidae	Mammals	Native	Southamerica	Lagomorphs

Pia 2013	12	533	*Lycalopex culpaeus*	Canidae	Mammals	Native	Southamerica	Lagomorphs
Pia 2013	29	371	*Puma concolor*	Felidae	Mammals	Native	Southamerica	Lagomorphs
Pia et al. 2003	13	277	*Lycalopex culpaeus*	Canidae	Mammals	Native	Southamerica	Lagomorphs
Pruett-Jones 1980	2	294	*Falco peregrinus*	Falconidae	Birds	Native	Oceania	Lagomorphs
Rau & Jimenez 2002	35	79	*Puma concolor*	Felidae	Mammals	Native	Southamerica	Lagomorphs
Risbey et al. 1999	38	194	*Vulpes vulpes*	Canidae	Mammals	Exotic	Oceania	Lagomorphs
Robertshaw & Harden 1985	127	2232	*Canis lupus dingo*	Canidae	Mammals	Exotic	Oceania	Lagomorphs
Robertshaw & Harden 1986	46	1590	*Canis lupus dingo*	Canidae	Mammals	Exotic	Oceania	Lagomorphs
Rubio et al. 2013	217	326	*Lycalopex culpaeus*	Canidae	Mammals	Native	Southamerica	Lagomorphs
Saggese & DeLucca 2001	36	57	*Geranoaetus melanoleucus*	Accipitridae	Birds	Native	Southamerica	Lagomorphs
Santander et al.	46	235	*Parabuteo unicinctus*	Accipitridae	Birds	Native	Southamerica	Lagomorphs
Schlatter et al. 1980	24	391	*Buteo polyosoma*	Accipitridae	Birds	Native	Southamerica	Lagomorphs
Schlatter et al. 1980	32	170	*Geranoaetus melanoleucus*	Accipitridae	Birds	Native	Southamerica	Lagomorphs
Sharp et al. 2002 (a)	475	1267	*Aquila audax*	Accipitridae	Birds	Native	Oceania	Lagomorphs
Sharp et al. 2002 (b)	970	2071	*Aquila audax*	Accipitridae	Birds	Native	Oceania	Lagomorphs
Silva & Croft 2007	63	192	*Aquila audax*	Accipitridae	Birds	Native	Oceania	Lagomorphs
Skewes et al. 2012	14	79	*Puma concolor*	Felidae	Mammals	Native	Southamerica	Lagomorphs
Smith et al. 1995	91	156	*Mustela furo*	Mustelidae	Mammals	Exotic	Oceania	Lagomorphs
Starker Leopold & Wolfe 1969	1582	2612	*Aquila audax*	Accipitridae	Birds	Native	Oceania	Lagomorphs
Starker Leopold & Wolfe 1969	396	2612	*Aquila audax*	Accipitridae	Birds	Native	Oceania	Lagomorphs
Travaini et al. 2001	403	2760	*Caracara plancus*	Falconidae	Birds	Native	Southamerica	Lagomorphs
Travaini et al. 2001	2	2760	*Caracara plancus*	Falconidae	Birds	Native	Southamerica	Lagomorphs*
Travaini et al. 2013	77	952	*Buteo polyosoma*	Accipitridae	Birds	Native	Southamerica	Lagomorphs
Trejo et al. 2005	27	663	*Bubo magellanicus*	Strigidae	Birds	Native	Southamerica	Lagomorphs
Trejo et al. 2005	6	1112	*Tyto alba*	Tytonidae	Birds	Native	Southamerica	Lagomorphs
Trejo et al. 2006 (a)	133	525	*Geranoaetus melanoleucus*	Accipitridae	Birds	Native	Southamerica	Lagomorphs
Trejo et al. 2006 (b)	1	597	*Buteo albigula*	Accipitridae	Birds	Native	Southamerica	Lagomorphs
Trejo y Lambertucci 2007	8	1889	*Tyto alba*	Tytonidae	Birds	Native	Southamerica	Lagomorphs

Trejo y Lambertucci 2007	2	2299	*Tyto alba*	Tytonidae	Birds	Native	Southamerica	Lagomorphs
Vargas et al. 2007	47	3033	*Caracara plancus*	Falconidae	Birds	Native	Southamerica	Lagomorphs
Walker et al. 2007	18	1030	*Leopardus colocolo*	Felidae	Mammals	Native	Southamerica	Lagomorphs
Walker et al. 2007	2	75	*Leopardus jacobitus*	Felidae	Mammals	Native	Southamerica	Lagomorphs
Walker et al. 2007	63	930	*Lycalopex culpaeus*	Canidae	Mammals	Native	Southamerica	Lagomorphs
White et al. 2006	93	503	*Vulpes vulpes*	Canidae	Mammals	Exotic	Oceania	Lagomorphs
Whitehouse 1977	8	261	*Canis lupus dingo*	Canidae	Mammals	Exotic	Oceania	Lagomorphs
Yañez et al. 1986	357	682	*Puma concolor*	Felidae	Mammals	Native	Southamerica	Lagomorphs
Zapata et al. 2005	104	316	*Lycalopex culpaeus*	Canidae	Mammals	Native	Southamerica	Lagomorphs
Zapata et al. 2005	15	203	*Lycalopex griseus*	Canidae	Mammals	Native	Southamerica	Lagomorphs
Zuñiga & Muñoz-Pedreros 2014	8	88	*Puma concolor*	Felidae	Mammals	Native	Southamerica	Lagomorphs
Zuñiga & Muñoz-Pedreros 2014	20	88	*Puma concolor*	Felidae	Mammals	Native	Southamerica	Lagomorphs*
Zuñiga et al. 2008	19	159	*Lycalopex griseus*	Canidae	Mammals	Native	Southamerica	Lagomorphs*
Zuñiga et al. 2008	2	159	*Lycalopex griseus*	Canidae	Mammals	Native	Southamerica	Lagomorphs
Astorga Saavedra 2013	9	50	*Oncifelis guigna*	Felidae	Mammals	Native	Southamerica	Native No Lagomorphs
Baladrón et al. 2006	60	213	*Buteo polyosoma*	Accipitriformes	Birds	Native	Southamerica	Native No Lagomorphs
Burton & Olsen 1997	4	363	*Accipiter fasciatus*	Accipitriformes	Birds	Native	Oceania	Native No Lagomorphs
Burton & Olsen 1997	47	467	*Accipiter novaehollandiae*	Accipitriformes	Birds	Native	Oceania	Native No Lagomorphs
Caneppucia et al. 2008	79	447	*Leopardus geofroyii*	Felidae	Mammals	Native	Southamerica	Native No Lagomorphs
Caneppucia et al. 2008	59	1854	*Lycalopex gymnocercus*	Canidae	Mammals	Native	Southamerica	Native No Lagomorphs
Cirignoli et al. 2001	122	223	*Asio flammeus*	Strigidae	Birds	Native	Southamerica	Native No Lagomorphs
De santis et al. 1994	93	304	*Tyto alba*	Tytonidae	Birds	Native	Southamerica	Native No Lagomorphs
Donadio et al. 2009	2	75	*Bubo magellanicus*	Strigidae	Birds	Native	Southamerica	Native No Lagomorphs
Donadio et al. 2009	52	392	*Tyto alba*	Tytonidae	Birds	Native	Southamerica	Native No Lagomorphs
Figueroa Rojas et al. 2003	74	105	*Buteo polyosoma*	Accipitriformes	Birds	Native	Southamerica	Native No Lagomorphs
Fitzsimons et al. 2014	7	21	*Aquila audax*	Accipitriformes	Birds	Native	Oceania	Native No Lagomorphs
Lapidge & Henshall 2001	1	38	*Vulpes vulpes*	Canidae	Mammals	Exotic	Oceania	Native No Lagomorphs
Leveau et al. 2006	893	3251	*Tyto alba*	Tytonidae	Birds	Native	Southamerica	Native No Lagomorphs

Marquet et al. 1993	39	185	*Lycalopex culpaeus*	Canidae	Mammals	Native	Southamerica	Native No Lagomorphs
Marquet et al. 1993	38	123	*Lycalopex griseus*	Canidae	Mammals	Native	Southamerica	Native No Lagomorphs
Martinez et al. 1998	275	812	*Asio flammeus*	Strigidae	Birds	Native	Southamerica	Native No Lagomorphs
Mella Avila 2002	10	42	*Bubo magellanicus*	Strigidae	Birds	Native	Southamerica	Native No Lagomorphs
Paltridge 2002	31	77	*Canis lupus dingo*	Canidae	Mammals	Exotic	Oceania	Native No Lagomorphs
Paltridge 2002	35	123	*Vulpes vulpes*	Canidae	Mammals	Exotic	Oceania	Native No Lagomorphs
Pokines 2007	153	280	*Bubo magellanicus*	Strigidae	Birds	Native	Southamerica	Native No Lagomorphs
Purdey et al. 2004	10	115	*Mustela erminea*	Mustelidae	Mammals	Exotic	Oceania	Native No Lagomorphs
Rickard 1996	11	201	*Mustela erminea*	Mustelidae	Mammals	Exotic	Oceania	Native No Lagomorphs
Sade et al. 2012	24	55	*Galictis cuja*	Mustelidae	Mammals	Native	Southamerica	Native No Lagomorphs
Salvador Jr. et al. 2008	1	41	*Geranoaetus melanoleucus*	Accipitriformes	Birds	Native	Southamerica	Native No Lagomorphs
Santillan et al. 2010	9	82	*Falco peregrinus*	Falconiformes	Birds	Native	Southamerica	Native No Lagomorphs
Teta & Contreras 2003	44	104	*Tyto alba*	Tytonidae	Birds	Native	Southamerica	Native No Lagomorphs
Teta et al. 2006	44	182	*Bubo magellanicus*	Strigidae	Birds	Native	Southamerica	Native No Lagomorphs
Trejo & Grigera 1998	186	755	*Bubo magellanicus*	Strigidae	Birds	Native	Southamerica	Native No Lagomorphs
Trejo & Guthmann 2003	237	1216	*Bubo magellanicus*	Strigidae	Birds	Native	Southamerica	Native No Lagomorphs
Allen et al. 2012	111	1460	*Canis lupus dingo*	Canidae	Mammals	Exotic	Oceania	Native with Lagomorphs
Arriagada et al. 2011	15	122	*Geranoaetus melanoleucus*	Accipitridae	Birds	Native	Southamerica	Native with Lagomorphs
Astorga Saavedra 2013	8	50	*Lycalopex culpaeus*	Canidae	Mammals	Native	Southamerica	Native with Lagomorphs
Aumann 2011	13	272	*Accipiter fasciatus*	Accipitridae	Birds	Native	Oceania	Native with Lagomorphs
Aumann 2011	25	119	*Aquila audax*	Accipitridae	Birds	Native	Oceania	Native with Lagomorphs
Aumann 2011	28	734	*Falco berigora*	Falconidae	Birds	Native	Oceania	Native with Lagomorphs
Aumann 2011	131	1826	*Hamirostra melanosternon*	Accipitridae	Birds	Native	Oceania	Native with Lagomorphs
Aumann 2011	46	597	*Hieraaetus morphnoides*	Accipitridae	Birds	Native	Oceania	Native with Lagomorphs
Baker-Gabb 1981	7	477	*Circus approximans*	Accipitridae	Birds	Native	Oceania	Native with Lagomorphs
Baker-Gabb 1983	4	126	*Accipiter fasciatus*	Accipitridae	Birds	Native	Oceania	Native with Lagomorphs
Baker-Gabb 1983	7	91	*Aquila audax*	Accipitridae	Birds	Native	Oceania	Native with Lagomorphs
Baker-Gabb 1983	64	355	*Circus assimilis*	Accipitridae	Birds	Native	Oceania	Native with Lagomorphs
Baker-Gabb 1983	12	246	*Falco berigora*	Falconidae	Birds	Native	Oceania	Native with Lagomorphs

Baker-Gabb 1983	2	177	*Falco cenchroides*	Falconidae	Birds	Native	Oceania	Native with Lagomorphs
Baker-Gabb 1983	59	335	*Falco subniger*	Falconidae	Birds	Native	Oceania	Native with Lagomorphs
Baker-Gabb 1983	14	159	*Haliastur sphenurus*	Accipitridae	Birds	Native	Oceania	Native with Lagomorphs
Baker-Gabb 1983	5	211	*Hamirostra melanosternon*	Accipitridae	Birds	Native	Oceania	Native with Lagomorphs
Baker-Gabb 1983	5	159	*Hieraaetus morphnoides*	Accipitridae	Birds	Native	Oceania	Native with Lagomorphs
Ballejo y De Santis 2013	6	1244	*Coragyps atratus*	Cathartidae	Birds	Native	Southamerica	Native with Lagomorphs
Belcher et al. 2007	183	414	*Dasyurus maculatus*	Dasyuridae	Mammals	Native	Oceania	Native with Lagomorphs
Biondi et al. 2005	14	4553	*Milvago chimango*	Falconidae	Birds	Native	Southamerica	Native with Lagomorphs
Birochio 2008	62	293	*Lycalopex gymnocercus*	Canidae	Mammals	Native	Southamerica	Native with Lagomorphs
Bisceglia et al. 2008	60	441	*Leopardus geofroyii*	Felidae	Mammals	Native	Southamerica	Native with Lagomorphs
Branch et al. 1996	251	335	*Puma concolor*	Felidae	Mammals	Native	Southamerica	Native with Lagomorphs
Brook y Kutt. 2011	23	184	*Canis lupus dingo*	Canidae	Mammals	Exotic	Oceania	Native with Lagomorphs
Brooker y Ridhpath 1980	81	3006	*Aquila audax*	Accipitridae	Birds	Native	Oceania	Native with Lagomorphs
Brunner et al. 1975	170	1888	*Vulpes vulpes*	Canidae	Mammals	Exotic	Oceania	Native with Lagomorphs
Catry et al. 2008	10	46	*Buteo polyosoma*	Accipitridae	Birds	Native	Southamerica	Native with Lagomorphs
Catry et al. 2008	240	406	*Phalcoboenus australis*	Falconidae	Birds	Native	Southamerica	Native with Lagomorphs
Cherrimann 2008	31	247	*Aquila audax*	Accipitridae	Birds	Native	Oceania	Native with Lagomorphs
Coates & Wright 2003	219	33	*Vulpes vulpes*	Canidae	Mammals	Exotic	Oceania	Native with Lagomorphs
Collins & Croft 2007	75	110	*Aquila audax*	Accipitridae	Birds	Native	Oceania	Native with Lagomorphs
Coman 1973	24	967	*Vulpes vulpes*	Canidae	Mammals	Exotic	Oceania	Native with Lagomorphs
Corbett y Newsome 1987	43	285	*Canis lupus dingo*	Canidae	Mammals	Exotic	Oceania	Native with Lagomorphs
Correa & Roa 2005	6	23	*Lycalopex culpaeus*	Canidae	Mammals	Native	Southamerica	Native with Lagomorphs
Correa & Roa 2005	3	17	*Lycalopex griseus*	Canidae	Mammals	Native	Southamerica	Native with Lagomorphs
Correa & Roa 2005	5	29	*Oncifelis guigna*	Felidae	Mammals	Native	Southamerica	Native with Lagomorphs
Dawson et al. 2007	79	1466	*Dasyurus maculatus*	Dasyuridae	Mammals	Native	Oceania	Native with Lagomorphs
Debus et al. 2007	3	49	*Aquila audax*	Accipitridae	Birds	Native	Oceania	Native with Lagomorphs
Donadio et al. 2010	64	538	*Puma concolor*	Felidae	Mammals	Native	Southamerica	Native with Lagomorphs
Donazar et al. 1997	211	1324	*Bubo magellanicus*	Strigidae	Birds	Native	Southamerica	Native with Lagomorphs
Ebensperger et al. 1991	5	34	*Galictis cuja*	Mustelidae	Mammals	Native	Southamerica	Native with Lagomorphs

Ebensperger et al. 1991	9	69	*Lycalopex culpaeus*	Canidae	Mammals	Native	Southamerica	Native with Lagomorphs
Ebensperger et al. 1991	25	134	*Tyto alba*	Strigidae	Birds	Native	Southamerica	Native with Lagomorphs
Elbroch y Wittmer 2013	332	433	*Puma concolor*	Felidae	Mammals	Native	Southamerica	Native with Lagomorphs
Farias & Kittlein 2008	32	1638	*Lycalopex gymnocercus*	Canidae	Mammals	Native	Southamerica	Native with Lagomorphs
Figueroa Rojas y Gonzales Acuña 2006	25	68	*Parabuteo unicinctus*	Accipitridae	Birds	Native	Southamerica	Native with Lagomorphs
Formoso et al. 2012	265	1637	*Bubo magellanicus*	Strigidae	Birds	Native	Southamerica	Native with Lagomorphs
Franklin et al. 1999	93	405	*Puma concolor*	Felidae	Mammals	Native	Southamerica	Native with Lagomorphs
Fuentes et al. 2005	3	39	*Haliastur sphenurus*	Accipitridae	Birds	Native	Oceania	Native with Lagomorphs
Fuentes et al. 2007	67	260	*Aquila audax*	Accipitridae	Birds	Native	Oceania	Native with Lagomorphs
Fuentes et al. 2007	25	330	*Aquila audax*	Accipitridae	Birds	Native	Oceania	Native with Lagomorphs
Galuppo Gaete 2014	24	51	*Oncifelis guigna*	Felidae	Mammals	Native	Southamerica	Native with Lagomorphs
Garcia & Kittlein 2005	72	469	*Lycalopex gymnocercus*	Canidae	Mammals	Native	Southamerica	Native with Lagomorphs
Glen & Dickman 2006	113	634	*Dasyurus maculatus*	Dasyuridae	Mammals	Native	Oceania	Native with Lagomorphs
Glen & Dickman 2008	113	424	*Dasyurus maculatus*	Dasyuridae	Mammals	Native	Oceania	Native with Lagomorphs
Glen et al. 2006	11	48	*Vulpes vulpes*	Canidae	Mammals	Exotic	Oceania	Native with Lagomorphs
Glen et al. 2010	60	392	*Dasyurus geofroyii*	Dasyuridae	Mammals	Native	Oceania	Native with Lagomorphs
Glen et al. 2011	11	68	*Canis lupus dingo*	Canidae	Mammals	Exotic	Oceania	Native with Lagomorphs
Glen et al. 2011	7	168	*Dasyurus maculatus*	Dasyuridae	Mammals	Native	Oceania	Native with Lagomorphs
Glen et al. 2011	8	168	*Dasyurus maculatus*	Dasyuridae	Mammals	Native	Oceania	Native with Lagomorphs
Glen et al. 2011	6	95	*Vulpes vulpes*	Canidae	Mammals	Exotic	Oceania	Native with Lagomorphs
Green & Osborne 1981	8	1159	*Vulpes vulpes*	Canidae	Mammals	Exotic	Oceania	Native with Lagomorphs
Hiraldo et al. 1995	34	1254	*Geranoaetus melanoleucus*	Accipitridae	Birds	Native	Southamerica	Native with Lagomorphs
Iriarte et al. 1989	105	371	*Lycalopex culpaeus*	Canidae	Mammals	Native	Southamerica	Native with Lagomorphs
Iriarte et al. 1990	32	162	*Bubo magellanicus*	Strigidae	Birds	Native	Southamerica	Native with Lagomorphs
Iriarte et al. 1990	1	93	*Geranoaetus melanoleucus*	Accipitridae	Birds	Native	Southamerica	Native with Lagomorphs
Iriarte et al. 1991	136	590	*Puma concolor*	Felidae	Mammals	Native	Southamerica	Native with Lagomorphs
Jaksic & Yañez 1980	21	114	*Bubo magellanicus*	Strigidae	Birds	Native	Southamerica	Native with Lagomorphs
Jaksic et al. 1980	111	172	*Parabuteo unicinctus*	Accipitridae	Birds	Native	Southamerica	Native with Lagomorphs

Jaksic et al. 1981	121	3038	*Athene cunicularia*	Strigidae	Birds	Native	Southamerica	Native with Lagomorphs
Jaksic et al. 1981	21	114	*Bubo magellanicus*	Strigidae	Birds	Native	Southamerica	Native with Lagomorphs
Jaksic et al. 1981	225	391	*Buteo polyosoma*	Accipitridae	Birds	Native	Southamerica	Native with Lagomorphs
Jaksic et al. 1981	95	164	*Geranoaetus melanoleucus*	Accipitridae	Birds	Native	Southamerica	Native with Lagomorphs
Jaksic et al. 1981	131	319	*Lycalopex culpaeus*	Canidae	Mammals	Native	Southamerica	Native with Lagomorphs
Jaksic et al. 1981	111	172	*Parabuteo unicinctus*	Accipitridae	Birds	Native	Southamerica	Native with Lagomorphs
Jaksic et al. 1986	72	339	*Bubo magellanicus*	Strigidae	Birds	Native	Southamerica	Native with Lagomorphs
Jaksic et al. 1990	40	932	*Lycalopex fulvipes*	Canidae	Mammals	Native	Southamerica	Native with Lagomorphs
Jarman et al. 2007	176	1023	*Dasyurus maculatus*	Dasyuridae	Mammals	Native	Oceania	Native with Lagomorphs
Jimenez & Jaksic 1988	3	1259	*Circus cinereus*	Accipitridae	Birds	Native	Southamerica	Native with Lagomorphs
Jimenez & Jaksic 1989	21	65	*Geranoaetus melanoleucus*	Accipitridae	Birds	Native	Southamerica	Native with Lagomorphs
Jimenez & Jaksic 1993	23	165	*Parabuteo unicinctus*	Accipitridae	Birds	Native	Southamerica	Native with Lagomorphs
Johnson & Franklin 1991	27	397	*Leopardus geofroyii*	Felidae	Mammals	Native	Southamerica	Native with Lagomorphs
Johnson & Franklin 1994	40	784	*Lycalopex culpaeus*	Canidae	Mammals	Native	Southamerica	Native with Lagomorphs
Johnson & Franklin 1994	115	851	*Lycalopex griseus*	Canidae	Mammals	Native	Southamerica	Native with Lagomorphs
King 1991	121	2522	*Mustela erminea*	Mustelidae	Mammals	Exotic	Oceania	Native with Lagomorphs
Kirkwood et al. 2005	58	115	*Vulpes vulpes*	Canidae	Mammals	Exotic	Oceania	Native with Lagomorphs
Lambertucci et al. 2009	3	517	*Vultur gryphus*	Cathartidae	Birds	Native	Southamerica	Native with Lagomorphs
Lugton 1991	8	404	*Vulpes vulpes*	Canidae	Mammals	Exotic	Oceania	Native with Lagomorphs
Lunney et al. 1990	204	613	*Vulpes vulpes*	Canidae	Mammals	Exotic	Oceania	Native with Lagomorphs
McDonald et al. 2012	14	307	*Falco berigora*	Falconidae	Birds	Native	Oceania	Native with Lagomorphs
Micthell & Banks 2005	57	307	*Vulpes vulpes*	Canidae	Mammals	Exotic	Oceania	Native with Lagomorphs
Molsher et al. 2000	96	757	*Vulpes vulpes*	Canidae	Mammals	Exotic	Oceania	Native with Lagomorphs
Monserrat et al. 2005	50	390	*Bubo magellanicus*	Strigidae	Birds	Native	Southamerica	Native with Lagomorphs
Monserrat et al. 2005	12	193	*Buteo polyosoma*	Accipitridae	Birds	Native	Southamerica	Native with Lagomorphs
Monserrat et al. 2005	4	516	*Geranoaetus melanoleucus*	Accipitridae	Birds	Native	Southamerica	Native with Lagomorphs
Monteverde & Piudo 2011	9	34	*Lycalopex culpaeus*	Canidae	Mammals	Native	Southamerica	Native with Lagomorphs
Muñoz & Maura 1990	8	44	*Lycalopex culpaeus*	Canidae	Mammals	Native	Southamerica	Native with Lagomorphs
Nabte et al. 2006	132	1232	*Bubo magellanicus*	Strigidae	Birds	Native	Southamerica	Native with Lagomorphs

Newsome 1984	204	1102	*Canis lupus dingo*	Canidae	Mammals	Exotic	Oceania	Native with Lagomorphs
Newsome et al. 2014	24	254	*Canis lupus dingo*	Canidae	Mammals	Exotic	Oceania	Native with Lagomorphs
Novaro et al. 2000	7	562	*Lycalopex culpaeus*	Canidae	Mammals	Native	Southamerica	Native with Lagomorphs
Novaro et al. 2000	2	61	*Lycalopex gymnocercus*	Canidae	Mammals	Native	Southamerica	Native with Lagomorphs
Novaro et al. 2000	1	99	*Puma concolor*	Felidae	Mammals	Native	Southamerica	Native with Lagomorphs
Olsen et al. 2006 (a)	3	22	*Accipiter fasciatus*	Accipitridae	Birds	Native	Oceania	Native with Lagomorphs
Olsen et al. 2006 (a)	10	52	*Falco peregrinus*	Falconidae	Birds	Native	Oceania	Native with Lagomorphs
Olsen et al. 2006 (b)	15	118	*Aquila audax*	Accipitridae	Birds	Native	Oceania	Native with Lagomorphs
Olsen et al. 2006 (b)	21	116	*Haliaeetus leucogaster*	Accipitridae	Birds	Native	Oceania	Native with Lagomorphs
Olsen et al. 2010	116	1421	*Aquila audax*	Accipitridae	Birds	Native	Oceania	Native with Lagomorphs
Olsen et al. 2010	10	192	*Hieraaetus morphnoides*	Accipitridae	Birds	Native	Oceania	Native with Lagomorphs
Olsen et al. 2014	16	629	*Aquila audax*	Accipitridae	Birds	Native	Oceania	Native with Lagomorphs
Palacios et al. 2012	10	178	*Leopardus geofroyii*	Felidae	Mammals	Native	Southamerica	Native with Lagomorphs
Palacios et al. 2012	12	422	*Lycalopex culpaeus*	Canidae	Mammals	Native	Southamerica	Native with Lagomorphs
Palacios et al. 2012	1	394	*Lycalopex gymnocercus*	Canidae	Mammals	Native	Southamerica	Native with Lagomorphs
Pavez et al. 1992	118	624	*Geranoaetus melanoleucus*	Accipitridae	Birds	Native	Southamerica	Native with Lagomorphs
Pessino et al. 2001	79	244	*Puma concolor*	Felidae	Mammals	Native	Southamerica	Native with Lagomorphs
Pia 2013	2	371	*Puma concolor*	Felidae	Mammals	Native	Southamerica	Native with Lagomorphs
Pia et al. 2003	24	277	*Lycalopex culpaeus*	Canidae	Mammals	Native	Southamerica	Native with Lagomorphs
Pruett-Jones 1980	50	294	*Falco peregrinus*	Falconidae	Birds	Native	Oceania	Native with Lagomorphs
Rau & Jimenez 2002	15	79	*Puma concolor*	Felidae	Mammals	Native	Southamerica	Native with Lagomorphs
Robertshaw & Harden 1985	608	2232	*Canis lupus dingo*	Canidae	Mammals	Exotic	Oceania	Native with Lagomorphs
Robertshaw & Harden 1986	660	1590	*Canis lupus dingo*	Canidae	Mammals	Exotic	Oceania	Native with Lagomorphs
Rubio et al. 2013	29	326	*Lycalopex culpaeus*	Canidae	Mammals	Native	Southamerica	Native with Lagomorphs
Saggese & DeLucca 2001	3	57	*Geranoaetus melanoleucus*	Accipitridae	Birds	Native	Southamerica	Native with Lagomorphs
Santander et al.	24	235	*Parabuteo unicinctus*	Accipitridae	Birds	Native	Southamerica	Native with Lagomorphs
Schlatter et al. 1980	225	391	*Buteo polyosoma*	Accipitridae	Birds	Native	Southamerica	Native with Lagomorphs
Schlatter et al. 1980	98	170	*Geranoaetus melanoleucus*	Accipitridae	Birds	Native	Southamerica	Native with Lagomorphs
Sharp et al. 2002 (a)	28	1267	*Aquila audax*	Accipitridae	Birds	Native	Oceania	Native with Lagomorphs

Sharp et al. 2002 (b)	12	2071	*Aquila audax*	Accipitridae	Birds	Native	Oceania	Native with Lagomorphs
Silva & Croft 2007	55	192	*Aquila audax*	Accipitridae	Birds	Native	Oceania	Native with Lagomorphs
Skewes et al. 2012	19	79	*Puma concolor*	Felidae	Mammals	Native	Southamerica	Native with Lagomorphs
Smith et al. 1995	6	156	*Mustela furo*	Mustelidae	Mammals	Exotic	Oceania	Native with Lagomorphs
Starker Leopold & Wolfe 1969	53	2612	*Aquila audax*	Accipitridae	Birds	Native	Oceania	Native with Lagomorphs
Travaini et al. 2001	24	2760	*Caracara plancus*	Falconidae	Birds	Native	Southamerica	Native with Lagomorphs
Travaini et al. 2013	80	952	*Buteo polyosoma*	Accipitridae	Birds	Native	Southamerica	Native with Lagomorphs
Trejo et al. 2005	127	663	*Bubo magellanicus*	Strigidae	Birds	Native	Southamerica	Native with Lagomorphs
Trejo et al. 2005	190	1112	*Tyto alba*	Tytonidae	Birds	Native	Southamerica	Native with Lagomorphs
Trejo et al. 2006 (a)	85	525	*Geranoaetus melanoleucus*	Accipitridae	Birds	Native	Southamerica	Native with Lagomorphs
Trejo et al. 2006 (b)	10	597	*Buteo albigula*	Accipitridae	Birds	Native	Southamerica	Native with Lagomorphs
Trejo y Lambertucci 2007	521	1889	*Tyto alba*	Tytonidae	Birds	Native	Southamerica	Native with Lagomorphs
Valenzuela et al. 2013	39	493	*Neovison vison*	Mustelidae	Mammals	Exotic	Southamerica	Native with Lagomorphs
Vargas et al. 2007	147	3033	*Caracara plancus*	Falconidae	Birds	Native	Southamerica	Native with Lagomorphs
Walker et al. 2007	55	1030	*Leopardus colocolo*	Felidae	Mammals	Native	Southamerica	Native with Lagomorphs
Walker et al. 2007	21	75	*Leopardus jacobitus*	Felidae	Mammals	Native	Southamerica	Native with Lagomorphs
Walker et al. 2007	30	930	*Lycalopex culpaeus*	Canidae	Mammals	Native	Southamerica	Native with Lagomorphs
White et al. 2006	135	503	*Vulpes vulpes*	Canidae	Mammals	Exotic	Oceania	Native with Lagomorphs
Whitehouse 1977	58	261	*Canis lupus dingo*	Canidae	Mammals	Exotic	Oceania	Native with Lagomorphs
Yañez et al. 1986	52	682	*Puma concolor*	Felidae	Mammals	Native	Southamerica	Native with Lagomorphs
Zapata et al. 2005	64	316	*Lycalopex culpaeus*	Canidae	Mammals	Native	Southamerica	Native with Lagomorphs
Zapata et al. 2005	40	203	*Lycalopex griseus*	Canidae	Mammals	Native	Southamerica	Native with Lagomorphs
Zuñiga & Muñoz-Pedreros 2014	21	88	*Puma concolor*	Felidae	Mammals	Native	Southamerica	Native with Lagomorphs
Zuñiga et al. 2008	51	159	*Lycalopex griseus*	Canidae	Mammals	Native	Southamerica	Native with Lagomorphs
Arriagada et al. 2011	5	122	*Geranoaetus melanoleucus*	Accipitridae	Birds	Native	Southamerica	Random preys
Aumann 1988	104	1766	*Accipiter fasciatus*	Accipitridae	Birds	Native	Oceania	Random preys
Aumann 2011	2	597	*Hieraaetus morphnoides*	Accipitridae	Birds	Native	Oceania	Random preys
Baker-Gabb 1983	3	91	*Aquila audax*	Accipitridae	Birds	Native	Oceania	Random preys

Belcher et al. 2007	5	414	*Dasyurus maculatus*	Dasyuridae	Mammals	Native	Oceania	Random preys
Biondi et al. 2005	1	4553	*Milvago chimango*	Falconidae	Birds	Native	Southamerica	Random preys
Birochio 2008	4	293	*Lycalopex gymnocercus*	Canidae	Mammals	Native	Southamerica	Random preys
Bustamante et al. 1997	129	1441	*Geranoaetus melanoleucus*	Accipitridae	Birds	Native	Southamerica	Random preys
Cherrimann 2008	12	247	*Aquila audax*	Accipitridae	Birds	Native	Oceania	Random preys
Correa & Roa 2005	2	29	*Oncifelis guigna*	Felidae	Mammals	Native	Southamerica	Random preys
Dawson et al. 2007	23	1466	*Dasyurus maculatus*	Dasyuridae	Mammals	Native	Oceania	Random preys
Debus 1984	1	101	*Hieraaetus morphnoides*	Accipitridae	Birds	Native	Oceania	Random preys
Donadio et al. 2010	2	538	*Puma concolor*	Felidae	Mammals	Native	Southamerica	Random preys
Donazar et al. 1997	33	1324	*Bubo magellanicus*	Strigidae	Birds	Native	Southamerica	Random preys
Elbroch y Wittmer 2013	7	433	*Puma concolor*	Felidae	Mammals	Native	Southamerica	Random preys
Farias & Kittlein 2008	34	1638	*Lycalopex gymnocercus*	Canidae	Mammals	Native	Southamerica	Random preys
Figueroa Rojas & Gonzalez Acuña 2006	1	68	*Parabuteo unicinctus*	Accipitridae	Birds	Native	Southamerica	Random preys
Formoso et al. 2012	226	1637	*Bubo magellanicus*	Strigidae	Birds	Native	Southamerica	Random preys
Fuentes et al. 1993	3	36	*Buteo polyosoma*	Accipitridae	Birds	Native	Southamerica	Random preys
Fuentes et al. 2007	11	260	*Aquila audax*	Accipitridae	Birds	Native	Oceania	Random preys
Glen & Dickman 2008	1	424	*Dasyurus maculatus*	Dasyuridae	Mammals	Native	Oceania	Random preys
Glen et al. 2010	45	392	*Dasyurus geofroyii*	Dasyuridae	Mammals	Native	Oceania	Random preys
Glen et al. 2011	7	168	*Dasyurus maculatus*	Dasyuridae	Mammals	Native	Oceania	Random preys
Hiraldo et al. 1995	6	1254	*Geranoaetus melanoleucus*	Accipitridae	Birds	Native	Southamerica	Random preys
Iriarte et al. 1989	1	614	*Lycalopex culpaeus*	Canidae	Mammals	Native	Southamerica	Random preys
Iriarte et al. 1990	13	162	*Bubo magellanicus*	Strigidae	Birds	Native	Southamerica	Random preys
Jaksic & Yañez 1980	5	114	*Bubo magellanicus*	Strigidae	Birds	Native	Southamerica	Random preys
Jaksic et al. 1990	184	932	*Lycalopex fulvipes*	Canidae	Mammals	Native	Southamerica	Random preys
Jimenez & Jaksic 1989	3	65	*Geranoaetus melanoleucus*	Accipitridae	Birds	Native	Southamerica	Random preys
Jimenez & Jaksic 1993	4	165	*Parabuteo unicinctus*	Accipitridae	Birds	Native	Southamerica	Random preys
Monserrat et al. 2005	2	193	*Buteo polyosoma*	Accipitridae	Birds	Native	Southamerica	Random preys
Monteverde & Piudo 2011	2	34	*Lycalopex culpaeus*	Canidae	Mammals	Native	Southamerica	Random preys

Novaro et al. 2000	8	526	*Lycalopex culpaeus*	Canidae	Mammals	Native	Southamerica	Random preys
Olsen et al. 2006 (a)	3	22	*Accipiter fasciatus*	Accipitridae	Birds	Native	Oceania	Random preys
Olsen et al. 2006 (a)	1	52	*Falco peregrinus*	Falconidae	Birds	Native	Oceania	Random preys
Olsen et al. 2010	20	1421	*Aquila audax*	Accipitridae	Birds	Native	Oceania	Random preys
Olsen et al. 2010	4	192	*Hieraaetus morphnoides*	Accipitridae	Birds	Native	Oceania	Random preys
Olsen et al. 2014	99	629	*Aquila audax*	Accipitridae	Birds	Native	Oceania	Random preys
Pavez et al. 1992	2	624	*Geranoaetus melanoleucus*	Accipitridae	Birds	Native	Southamerica	Random preys
Pessino et al. 2001	2	244	*Puma concolor*	Felidae	Mammals	Native	Southamerica	Random preys
Pia 2013	2	371	*Puma concolor*	Felidae	Mammals	Native	Southamerica	Random preys
Pia et al. 2003	18	277	*Lycalopex culpaeus*	Canidae	Mammals	Native	Southamerica	Random preys
Saggese & DeLucca 2001	5	57	*Geranoaetus melanoleucus*	Accipitridae	Birds	Native	Southamerica	Random preys
Schlatter et al. 1980	10	391	*Buteo polyosoma*	Accipitridae	Birds	Native	Southamerica	Random preys
Sharp et al. 2002 (a)	3	1267	*Aquila audax*	Accipitridae	Birds	Native	Oceania	Random preys
Travaini et al. 2001	3	2760	*Caracara plancus*	Falconidae	Birds	Native	Southamerica	Random preys
Vargas et al. 2007	39	3033	*Caracara plancus*	Falconidae	Birds	Native	Southamerica	Random preys
Walker et al. 2007	74	930	*Lycalopex culpaeus*	Canidae	Mammals	Native	Southamerica	Random preys
Yañez et al. 1986	9	682	*Puma concolor*	Felidae	Mammals	Native	Southamerica	Random preys
Zapata et al. 2005	10	316	*Lycalopex culpaeus*	Canidae	Mammals	Native	Southamerica	Random preys

(Starker Leopole & Wolfe 1970; Brooker & Ridpath 1980; Jaksić & Yáñez 1980; Pruett-Jones et al. 1980; Schlatter et al. 1980; Jaksić et al. 1980, 1990; Baker-Gabb 1981, 1981, 1984a, 1984b; Jaksic et al. 1981, 1986; Debus 1984; Yáñez et al. 1986; Jiménez & Jaksic 1988, 1993; Aumann 1988, 2001; Iriarte et al. 1989, 1989, 1990, 1991; Jiménez & Jaksić 1989; Muñoz et al. 1990; Johnson & Franklin 1991, 1994; Fuentes et al. 1993, 2005, 2007; Hiraldo et al. 1995; Branch et al. 1996; Bustamante et al. 1997; Donázar et al. 1997;

Franklin et al. 1999; Novaro et al. 2000; Pessino et al. 2001; Saggese & De Lucca 2001; Travaini et al. 2001, 2012; Rau & Jiménez 2002; Sharp et al. 2002a, 2002b; McDonald et al. 2003, 2006, 2012; Pia et al. 2003; McDonald 2004; Biondi et al. 2005; Correa & Roa 2005; García & Kittlein 2005; Monserrat et al. 2005; Trejo et al. 2005, 2006a, 2006b; Zapata et al. 2005; Glen & Dickman 2006, 2008; Nabte et al. 2006; Olsen et al. 2006a, 2006b, 2010, 2013, 2014; Figueroa & González-Acuña 2006; Belcher et al. 2007; Cherriman 2007; Collins & Croft 2007; Dawson et al. 2007; Debus et al. 2007; Jarman et al. 2007; Silva & Croft 2007; Trejo & Lambertucci 2007; Walker et al. 2007; Vargas et al. 2007; Bisceglia et al. 2008; Farias & Kittlein 2008; Zúñiga et al. 2008; Catry et al. 2008; Lambertucci et al. 2009, 2009; Donadio et al. 2010; Ellis et al. 2010; Glen et al. 2010, 2011; Arriagada et al. 2011; Castillo et al. 2011; Monteverde & Piudo 2011; Santander et al. 2011; Palacios et al. 2012; Skewes et al. 2012; Formoso et al. 2012; Pia 2013; Elbroch & Wittmer 2013; Ballejo et al. 2013; Galuppo Gaete 2014; Zúñiga & Muñoz-Pedreros 2014; Birochio 2015)

(McIntosh 1963; Coman 1973; Brunner et al. 1975; Whitehouse 1977; Green & Osborne 1981; Newsome et al. 1983, 2014; Robertshaw & Harden 1985, 1986; Corbett & Newsome 1987; Lunney et al. 1990; Ebensperger et al. 1991; King 1991; Lugton 1993; Marquet et al. 1993; De Santis et al. 1994; Murphy & Dowding 1994, 1995; Smith et al. 1995; Rickard 1996; Alterio & Moller 1997; Burton & Olsen 1997; Diuk-Wasser & Cassini 1998; Gillies & others 1998; Martinez et al. 1998; Trejo & Grigera 1998; Morley 1999; Short et al. 1999; Molsher et al. 2000; Cirignoli et al. 2001; Lapidge & Henshall 2001; Mella 2002; Paltridge 2002; Coates & Wright 2003; Figueroa Rojas et al. 2003; Trejo & Guthmann 2003; Purdey et al. 2004; Kirkwood et al. 2005; Mitchell & Banks 2005; Murphy et al. 2005, 2008; Baladrón et al. 2006; Glen et al. 2006, 2011; Leveau et al. 2006; Teta et al. 2006; White et al. 2006; Pokines 2007; Canepuccia et al. 2008; Donadio et al. 2009; Santillán et al. 2010; Brook & Kutt 2011; Allen et al. 2012; Sade et al. 2012; Astorga Saavedra 2013; Salvador-Jr et al. 2013; Valenzuela et al. 2013; Fitzsimons et al. 2014; Marlow et al. 2014)

Allen L, Goullet M, Palmer R. 2012. The diet of the dingo (*Canis lupus dingo and hybrids*) in north-eastern Australia: a supplement to the paper of Brook and Kutt (2011). The Rangeland Journal **34**:211–217.

Alterio N, Moller H. 1997. Diet of feral house cats *Felis catus*, ferrets *Mustela furo* and stoats *M. erminea* in grassland surrounding yellow-eyed penguin *Megadyptes antipodes* breeding areas, South Island, New Zealand. Journal of Zoology **243**:869–877.

Arriagada AM, Arriagada JL, Baessolo LA, Suazo CG. 2011. Dieta estival del águila (Geranoaetus melanoleucus) en la región de Aysén, Patagonia Chilena. Ecotropicos **24**:2.

Aumann T. 1988. The Diet of the Brown Goshawk, *Accipiter-Fasciatus*, in Southeastern Australia. Wildlife Research **15**:587–594.

Aumann T. 2001. An intraspecific and interspecific comparison of raptor diets in the south-west of the Northern Territory, Australia. Wildlife Research **28**:379–393.

Baker-Gabb DJ. 1981. The diet of the Australasian harrier (*Circus approximans*) in the Manawatu-Rangitikei sand country, New Zealand. Notornis **28**:241–254.

Baker-Gabb DJ. 1984a. The breeding ecology of twelve species of diurnal raptor in north-western Victoria. Wildlife Research **11**:145–160.

Baker-Gabb DJ. 1984b. The feeding ecology and behaviour of seven species of raptor overwintering in coastal Victoria. Wildlife Research **11**:517–532.

Baladrón AV, Bó MS, Malizia AI. 2006. Winter diet and time-activity budgets of the Red-Backed Hawk (*Buteo polyosoma*) in the coastal grasslands of Buenos Aires province, Argentina. Journal of Raptor Research **40**:65–70.

Ballejo F, Santis D, M LJ. 2013. Dieta estacional del Jote Cabeza Negra (*Coragyps atratus*) en un área rural y una urbana en el noroeste patagónico. El hornero **28**:07–14.

Belcher CA, Nelson JL, Darrant JP. 2007. Diet of the tiger quoll (*Dasyurus maculatus*) in south-eastern Australia. Australian Journal of Zoology **55**:117–122.

Biondi LM, Bó MS, Favero M. 2005. Dieta del chimango (*Milvago chimango*) durante el periodo reproductivo en el sudeste de la provincia de Buenos Aires, Argentina. Ornitología Neotropical **16**:31–42.

Birochio D. 2015. Ecología trófica de *Lycalopex gymnocercus* en la región pampeana: Un acercamiento inferencial al uso de los recursos. Available from http://repositoriodigital.uns.edu.ar/handle/123456789/2311 (accessed November 7, 2015).

Bisceglia SB, Pereira JA, Teta P, Quintana RD. 2008. Food habits of Geoffroy’s cat (*Leopardus geoffroyi*) in the central Monte desert of Argentina. Journal of Arid Environments **72**:1120–1126.

Branch LC, Pessino M, Villarreal D. 1996. Response of pumas to a population decline of the plains vizcacha. Journal of Mammalogy **77**:1132–1140.

Brooker MG, Ridpath MG. 1980. The diet of the Wedge-Tailed Eagle, *Aquila audax*, in western Australia. Wildlife research **7**:433–452.

Brook LA, Kutt AS. 2011. The diet of the dingo (*Canis lupus dingo*) in north-eastern Australia with comments on its conservation implications. The Rangeland Journal **33**:79–85.

Brunner H, Lloyd JW, Coman BJ. 1975. Fox Scat Analysis in a Forest Park in South-Eastern Australia. Wildlife Research **2**:147–154.

Burton AM, Olsen P. 1997. Niche partitioning by two sympatric goshawks in the Australian wet tropics: breeding-season diet. Wildlife Research **24**:45–52.

Bustamante J, Donázar JA, Hiraldo F, Ceballos O, Travaini A. 1997. Differential habitat selection by immature and adult Grey Eagle-buzzards *Geranoaetus melanoleucus*. Ibis **139**:322–330.

Canepuccia AD, Farias AA, Escalante AH, Iribarne O, Novaro A, Isacch JP. 2008. Differential responses of marsh predators to rainfall-induced habitat loss and subsequent variations in prey availability. Canadian Journal of Zoology **86**:407–418.

Castillo DF, Birochio DE, Lucherini M, Casanave EB. 2011. Diet of adults and cubs of Lycalopex gymnocercus in Pampas grassland: a validation of the Optimal Foraging Theory? Pages 251–256 Annales Zoologici Fennici. BioOne. Available from http://www.bioone.org/doi/abs/10.5735/086.048.0406 (accessed November 7, 2015).

Catry P, Lecoq M, Strange IJ. 2008. Population growth and density, diet and breeding success of striated caracaras *Phalcoboenus australis* on New Island, Falkland Islands. Polar Biology **31**:1167–1174.

Cherriman SC. 2007. Territory size and diet throughout the year of the Wedge-tailed Eagle *Aquila audax* in the Perth region, Western Australia. B. Sc.(Hons) Thesis, Curtin University, Western Australia. Available from http://www.simoncherriman.com/Simon_Cherriman/Research_files/HONOURS%20THESIS.pdf (accessed November 7, 2015).

Cirignoli S, Podestá DH, Pardiñas UFJ. 2001. Diet of the Short-eared Owl in northwestern Argentina. Journal of Raptor Research **35**:68–69.

Coates TD, Wright CJ. 2003. Predation of southern brown bandicoots *Isoodon obesulus* by the European red fox *Vulpes vulpes* in south-east Victoria. Australian Mammalogy **25**:107–110.

Collins L, Croft DB. 2007. Factors influencing chick survival in the Wedge-tailed Eagle Aquila audax. Corella **31**:32–40.

Coman BJ. 1973. The diet of red foxes, *Vulpes vulpes* L., in Victoria. Australian Journal of Zoology **21**:391–401.

Corbett LK, Newsome AE. 1987. The feeding ecology of the dingo. Oecologia **74**:215–227.

Correa P, Roa A. 2005. Relaciones tróficas entre *Oncifelis guigna*, *Lycalopex culpaeus*, *Lycalopex griseus* y *Tyto alba* en un ambiente fragmentado de la zona central de Chile. Mastozoología neotropical **12**:57–60.

Dawson JP, Claridge AW, Triggs B, Paull DJ. 2007. Diet of a native carnivore, the spotted-tailed quoll (*Dasyurus maculatus*), before and after an intense wildfire. Wildlife Research **34**:342–351.

Debus SJ. 1984. Biology of the little eagle on the northern tablelands of New South Wales. Emu **84**:87–92.

Debus SJS, Hatfield TS, Ley AJ, Rose AB, others. 2007. Breeding biology and diet of the Wedge-tailed Eagle *Aquila audax* in the New England region of New South Wales. Australian Field Ornithology **24**:93–120.

De Santis LJ, Basso NG, Noriega JI, Grossman MF. 1994. Explotación del recurso trófico por la lechuza de los campanarios (*Tyto alba*) en el oeste de Chubut, Argentina. Studies on neotropical fauna and environment **29**:43–47.

Diuk-Wasser MA, Cassini MH. 1998. A study on the diet of minor grisons and a preliminary analysis of their role in the control of rabbits in Patagonia. Studies on Neotropical Fauna and Environment **33**:3–6.

Donadio E, Merino ML, Bolgeri MJ. 2009. Diets of two coexisting owls in the High Andes of Northwestern Argentina. Ornitología Neotropical **20**. Available from http://www.rufford.org/files/Ornitologia%20Neotropical%2020.pdf (accessed November 9, 2015).

Donadio E, Novaro AJ, Buskirk SW, Wurstten A, Vitali MS, Monteverde MJ. 2010. Evaluating a potentially strong trophic interaction: pumas and wild camelids in protected areas of Argentina. Journal of Zoology **280**:33–40.

Donázar JA, Travaini A, Ceballos O, Delibes M, Hiraldo F, others. 1997. Food habits of the great horned owl in northwestern Argentine Patagonia: the role of introduced lagomorphs. Journal of Raptor Research **31**:364–369.

Ebensperger LA, Mella JE, Simonetti JA. 1991. Trophic-niche relationships among *Galictis cuja*, *Dusicyon culpaeus*, and *Tyto alba* in central Chile. Journal of Mammalogy **72**:820–823.

Elbroch LM, Wittmer HU. 2013. The effects of puma prey selection and specialization on less abundant prey in Patagonia. Journal of Mammalogy **94**:259–268.

Ellis DH, Saggese MD, Caballero I, Wayne N, Trejo A, Seijas S, Barbar F. 2010. The Pallid Color Morph of *Falco peregrinus cassini*: A History of Former Studies, a Summary of Recent work, and Projections for Future Work. Available from https://www.academia.edu/1713682/The_Pallid_Color_Morph_of_Falco_peregrinus_cassini_A_History_of_Former_Studies_a_Summary_of_Recent_work_and_Projections_for_Future_Work (accessed November 7, 2015).

Farias AA, Kittlein MJ. 2008. Small-scale spatial variability in the diet of pampas foxes (*Pseudalopex gymnocercus*) and human-induced changes in prey base. Ecological Research **23**:543–550.

Figueroa RAR, González-Acuña D. 2006. Prey of the harris's hawk (*Parabuteo unicinctus*) in a suburban area of southern chile. Journal of Raptor Research **40**:164–168.

Figueroa Rojas R A, Corales Stappung S E, Alvarado SA. 2003. Diet of the Red-backed Hawk (*Buteo polyosoma*) in a forested area of the Chilean Patagonia and its relation to the abundance of rodent prey. Hornero **18**:43–52.

Fitzsimons JA, Carlyon K, Thomas JL, Rose AB. 2014. The breeding diet of Wedge-tailed Eagles *Aquila audax* in the absence of rabbits: Kangaroo Island, South Australia. Corella **38 (1)**. Available from http://www.researchgate.net/profile/James_Fitzsimons/publication/260789352_The_breeding_diet_of_Wedge-tailed_Eagles_Aquila_audax_in_the_absence_of_rabbits_Kangaroo_Island_South_Australia/links/004635323686cb0834000000.pdf (accessed November 9, 2015).

Formoso AE, Pablo T, Germán C. 2012. Food Habits of the Magellanic Horned Owl (*Bubo virginianus magellanicus*) at Southernmost Patagonia, Argentina. Journal of Raptor Research **46**:401–406.

Franklin WL, Johnson WE, Sarno RJ, Iriarte JA. 1999. Ecology of the Patagonia puma *Felis concolor* patagonica in southern Chile. Biological Conservation **90**:33–40.

Fuentes E, Olsen J, Rose AB. 2005. Breeding diet at two Whistling Kite nests near Canberra. Australian Field Ornithology **22**:122–125.

Fuentes E, Olsen J, Rose AB. 2007. Diet, occupancy and breeding performance of wedge-tailed eagles *Aquila audax* near Canberra, Australia 2002-2003= four decades after Leopold and Wolfe. Available from http://citeseerx.ist.psu.edu/viewdoc/summary?doi=10.1.1.500.9813 (accessed November 7, 2015).

Fuentes MA, Simonetti JA, Sepulveda MS, Acevedo PA. 1993. Diet of Red-backed Buzzard (*Buteo polyosoma exsul*) and Short-eared Owl (*Asio flammeus suinda*) in the Juan Fernandez Archipelago off Chile. J. Raptor Res **27**:167–169.

Galuppo Gaete SE. 2014. Diet and activity patterns of Leopardus guigna in relation to prey availability in forest fragments of the Chilean temperate rainforest. UNIVERSITY OF MINNESOTA. Available from http://conservancy.umn.edu/handle/11299/167290 (accessed November 7, 2015).

García VB, Kittlein MJ. 2005. Diet, habitat use, and relative abundance of pampas fox (*Pseudalopex gymnocercus*) in northern Patagonia, Argentina. Mammalian Biology-Zeitschrift für Säugetierkunde **70**:218–226.

Gillies C, others. 1998. Aspects of the ecology and management of small mammalian predators in northern New Zealand. ResearchSpace@ Auckland. Available from https://researchspace.auckland.ac.nz/handle/2292/1942 (accessed February 23, 2016).

Glen AS, Dickman CR. 2006. Diet of the spotted-tailed quoll (*Dasyurus maculatus*) in eastern Australia: effects of season, sex and size. Journal of Zoology **269**:241–248.

Glen AS, Dickman CR. 2008. Niche overlap between marsupial and eutherian carnivores: does competition threaten the endangered spotted-tailed quoll? Journal of Applied Ecology **45**:700–707.

Glen AS, Fay AR, Dickman CR. 2006. Diets of sympatric red foxes *Vulpes vulpes* and wild dogs Canis lupus in the Northern Rivers Region, New South Wales. Australian Mammalogy **28**:101–104.

Glen AS, Pennay M, Dickman CR, Wintle BA, Firestone KB. 2011. Diets of sympatric native and introduced carnivores in the Barrington Tops, eastern Australia. Austral Ecology **36**:290–296.

Glen AS, Wayne A, Maxwell M, Cruz J. 2010. Comparative diets of the chuditch, a threatened marsupial carnivore, in the northern and southern jarrah forests, Western Australia. Journal of Zoology **282**:276–283.

Green K, Osborne WS. 1981. The Diet of Foxes, *Vulpes Vulpes* (L.), In Relation to Abundance of Prey Above the Winter Snowline in New South Wales. Wildlife Research **8**:349–360.

Hiraldo F, Donázar JA, Bustamante J, Ceballos O, Travaini A, Funes M. 1995. Breeding biology of a grey eagle-buzzard population in Patagonia. The Wilson Bulletin:675–685.

Iriarte JA, Franklin WL, Johnson WE. 1990. Diets of sympatric raptors in southern Chile. Journal of Raptor Research **24**:41–46.

Iriarte JA, Jimenez JE, Contreras LC, Jaksić FM. 1989. Small-mammal availability and consumption by the fox, *Dusicyon culpaeus*, in central Chilean scrublands. Journal of Mammalogy:641–645.

Iriarte JA, Johnson WE, Franklin WL. 1991. Feeding ecology of the Patagonia puma in southernmost Chile. Revista Chilena de Historia Natural **64**:145–156.

Jaksic FM, Greene HW, Yáñez JL. 1981. The guild structure of a community of predatory vertebrates in central Chile. Oecologia **49**:21–28.

Jaksić FM, Jiménez JE, Medel RG, Marquet PA. 1990. Habitat and diet of Darwin’s fox (*Pseudalopex fulvipes*) on the Chilean mainland. Journal of Mammalogy:246–248.

Jaksić FM, Yáñez JL. 1980. Differential utilization of prey resources by Great Horned Owls and Barn Owls in central Chile. The Auk:895–896.
Jaksic FM, Yáñez JL, Rau JR. 1986. Prey and trophic ecology of Great Horned Owls in western South America: an indication of latitudinal trends. Journal of Raptor Research **20**:113–116.
Jaksić FM, Yáñez JL, Schlatter RP. 1980. Prey of the Harris' Hawk in Central Chile. The Auk **97**:196–198.
Jarman PJ, Allen LR, Boschma DJ, Green SW. 2007. Scat contents of the spotted-tailed quoll *Dasyurus maculatus* in the New England gorges, north-eastern New South Wales. Australian journal of zoology **55**:63–72.
Jiménez JE, Jaksic FM. 1988. Ecology and behavior of southern South American Cinereous harriers, *Circus cinereus*. Revista Chilena de Historia Natural **61**:199–208.
Jiménez JE, Jaksić FM. 1989. Behavioral ecology of grey eagle-buzzards, *Geranoaetus melanoleucus,* in central Chile. Condor:913–921.
Jiménez JE, Jaksic FM. 1993. Observations on the comparative behavioral ecology of Harris' Hawk in central Chile. Journal of Raptor Research **27**:143–148.
Johnson WE, Franklin WL. 1991. Feeding and spatial ecology of *Felis geoffroyi* in southern Patagonia. Journal of Mammalogy:815–820.
Johnson WE, Franklin WL. 1994. Role of body size in the diets of sympatric gray and culpeo foxes. Journal of Mammalogy **75**:163–174.
King CM. 1991. Body size–prey size relationships in European stoats *Mustela erminea*: a test case. Ecography **14**:173–185.
Kirkwood R, Dann P, Belvedere M. 2005. A comparison of the diets of feral cats *Felis catus* and red foxes *Vulpes vulpes* on Phillip Island, Victoria. Australian Mammalogy **27**:89–93.
Lambertucci SA, Trejo A, Di Martino S, Sánchez-Zapata JA, Donázar JA, Hiraldo F. 2009. Spatial and temporal patterns in the diet of the Andean condor: ecological replacement of native fauna by exotic species. Animal Conservation **12**:338–345.
Lapidge SJ, Henshall S. 2001. Diet Of Foxes And Cats, With Evidence Of Predation On Yellow-Footed Rock-Wallabies (Petrogale Xanthopus Celeris) By Foxes In Southwsetern Queensland. Australian Mammalogy **23**:47–52.
Leveau LM, Teta P, Bogdaschewsky R, Pardiñas UF. 2006. Feeding habits of the Barn Owl (*Tyto alba*) along a longitudinal-latitudinal gradient in central Argentina. Ornitología Neotropical **17**:353–362.
Lugton IW. 1993. Diet of red foxes (*Vulpes vulpes*) in South-west New South Wales, with relevance to lamb predation. The Rangeland Journal **15**:39–47.
Lunney D, Triggs B, Eby P, Ashby E. 1990. Analysis of Scats of Dogs *Canis familiaris* and Foxes *Vulpes vulpes* (Canidae, Carnivora) in Coastal Forests Near Bega, New-South-Wales. Wildlife Research **17**:61–68.
Marlow NJ, Thomas ND, Williams AAE, Macmahon B, Lawson J. 2014. The diet of foxes (*Vulpes vulpes*) in fragmented Wheatbelt reserves in Western Australia: implications for woylies (*Bettongia penicillata*) and other native fauna. Conservation Science

Western Australia. Available from https://www.researchgate.net/profile/Neil_Thomas9/publication/280093181_The_diet_of_foxes_Vulpes_vulpes_in_fragmented_Wheatbelt_reserves_in_Western_Australia_implications_for_woylies_Bettongia_penicillata_and_other_native_fauna/links/55a854f308aea994671ddbfa.pdf (accessed February 23, 2016).

Marquet PA, Contreras LC, TORRESMURA J, Silva SI, Jaksic FM. 1993. Food habits of *Pseudalopex foxes* in the Atacama desert, pre-Andean ranges, and the high-Andean plateau of northernmost Chile. Mammalia **57**:131–135.

Martinez DR, Figueroa RA, Ocampo CL, Jaksic FM. 1998. Food habits and hunting ranges of short-eared owls (*Asio flammeus*) in agricultural landscapes of southern Chile. Journal of Raptor Research **32**:111–115.

McDonald PG. 2004. The breeding ecology and behaviour of a colour-marked population of Brown Falcons (*Falco berigora*). Emu **104**:1–6.

McDonald PG, Baker-Gabb D, Warkentin I. 2006. The breeding diet of different Brown Falcon (*Falco berigora*) pairs occupying the same territory over twenty years apart. Journal of Raptor Research **40**:228–231.

McDonald PG, Olsen J, Rose AB. 2012. The Diet of Breeding Brown Falcons (*Falco berigora*) In the Canberra Region, Australia, With Comparisons To Other Regions. Journal of Raptor Research **46**:394–400.

McDonald PG, Olsen PD, Baker-Gabb DJ. 2003. Territory fidelity, reproductive success and prey choice in the brown falcon, *Falco berigora*: a flexible bet-hedger? Australian Journal of Zoology **51**:399–414.

McIntosh DL. 1963. Food of the fox in the Canberra district. Wildlife Research **8**:1–20.

Mella J. 2002. Dieta del cernícalo (*Falco sparverius*) y del Tucúquere (*Bubo magellanicus*) en un ambiente cordillerano de Chile central. Boletín Chileno de Ornitología **9**:34–37.

Mitchell BD, Banks PB. 2005. Do wild dogs exclude foxes? Evidence for competition from dietary and spatial overlaps. Austral Ecology **30**:581–591.

Molsher RL, Gifford EJ, McIlroy JC. 2000. Temporal, spatial and individual variation in the diet of red foxes (*Vulpes vulpes*) in central New South Wales. Wildlife Research **27**:593–601.

Monserrat AL, Funes MC, Novaro AJ. 2005. Dietary response of three raptor species to an introduced prey in Patagonia. Revista Chilena de Historia Natural **78**:129–143.

Monteverde MJ, Piudo L. 2011. Activity Patterns of the Culpeo Fox (*Lycalopex culpaeus magellanica*) in a Non-Hunting Area of Northwestern Patagonia, Argentina. Mammal Study **36**:119–125.

Morley CG. 1999. The ecology and behaviour of feral ferrets (*Mustela furo*) in Canterbury farmland, New Zealand. Available from http://ir.canterbury.ac.nz/handle/10092/5824 (accessed February 23, 2016).

Muñoz A, Murúa R, others. 1990. Control of small mammals in a pine plantation (Central Chile) by modification of the habitat of predators (*Tyto alba*, Strigiforme and *Pseudalopex* sp., Canidae). Acta oecologica **11**:251–261.

Murphy EC, Dowding JE. 1994. Range and diet of stoats (*Mustela erminea*) in a New Zealand beech forest. New Zealand Journal of Ecology:11–18.

Murphy EC, Dowding JE. 1995. Ecology of the stoat in Nothofagus forest: home range, habitat use and diet at different stages of the beech mast cycle. New Zealand journal of ecology:97–109.

Murphy EC, Keedwell RJ, Brown KP, Westbrooke I. 2005. Diet of mammalian predators in braided river beds in the central South Island, New Zealand. Wildlife Research **31**:631–638.

Murphy E, Maddigan F, Edwards B, Clapperton K. 2008. Diet of stoats at Okarito Kiwi Sanctuary, South Westland, New Zealand. New Zealand Journal of Ecology:41–45.

Nabte MJ, Saba SL, Pardiñas UF. 2006. Dieta del Búho Magallánico (*Bubo magellanicus*) en el desierto del monte y la Patagonia argentina. Ornitología Neotropical **17**:27–38.

Newsome AE, Catling PC, Corbett LK. 1983. The feeding ecology of the dingo II. Dietary and numerical relationships with fluctuating prey populations in south-eastern Australia. Australian Journal of Ecology **8**:345–366.

Newsome TM, Ballard G-A, Crowther MS, Fleming PJ, Dickman CR. 2014. Dietary niche overlap of free-roaming dingoes and domestic dogs: the role of human-provided food. Journal of Mammalogy **95**:392–403.

Novaro AJ, Funes MC, Susan Walker R. 2000. Ecological extinction of native prey of a carnivore assemblage in Argentine Patagonia. Biological Conservation **92**:25–33.

Olsen J, Cooke B, Trost S, Judge D. 2014. Is wedge-tailed eagle, *Aquila audax*, survival and breeding success closely linked to the abundance of European rabbits, *Oryctolagus cuniculus*? Wildlife Research **41**:95–105.

Olsen J, Debus SJS, Rose AB, Judge D. 2013. Diets of White-bellied Sea-Eagles *Haliaeetus leucogaster* and Whistling Kites *Haliastur sphenurus* breeding near Canberra, 2003–2008. Corella. Available from http://www.researchgate.net/profile/Jerry_Olsen/publication/263297397_Diets_of_White-bellied_Sea-Eagles_Haliaeetus_leucogaster_and_Whistling_Kites_Haliastur_sphenurus_breeding_near_Canberra_20032008/links/00b7d53a7f557b231f000000.pdf (accessed November 9, 2015).

Olsen J, Fuentes E, Rose AB. 2006a. Trophic relationships between neighbouring White-bellied Sea-Eagles (*Haliaeetus leucogaster*) and Wedge-tailed Eagles (Aquila audax) breeding on rivers and dams near Canberra. Emu **106**:193–201.

Olsen J, Fuentes E, Rose AB, Trost S, others. 2006b. Food and hunting of eight breeding raptors near Canberra, 1990-1994. Available from http://search.informit.com.au/documentSummary;dn=307456379754589;res=IELHSS (accessed November 7, 2015).

Olsen J, Judge D, Fuentes E, Rose AB, Debus SJS. 2010. Diets of Wedge-tailed Eagles (*Aquila audax*) and Little Eagles (*Hieraaetus morphnoides*) Breeding Near Canberra, Australia. Journal of Raptor Research **44**:50–61.

Palacios R, Walker RS, Novaro AJ. 2012. Differences in diet and trophic interactions of Patagonian carnivores between areas with mostly native or exotic prey. Mammalian Biology - Zeitschrift für Säugetierkunde **77**:183–189.

Paltridge R. 2002. The diets of cats, foxes and dingoes in relation to prey availability in the Tanami Desert, Northern Territory. Wildlife Research **29**:389–403.

Pessino ME, Sarasola JH, Wander C, Besoky N. 2001. Respuesta a largo plazo del puma (*Puma concolor*) a una declinación poblacional de la vizcacha (*Lagostomus maximus*) en el desierto del Monte, Argentina. Ecología austral **11**:61–67.

Pia M, López MS, Novaro AJ. 2003. Effects of livestock on the feeding ecology of endemic culpeo foxes (*Pseudalopex culpaeus smithersi*) in central Argentina. Revista Chilena de Historia Natural **76**:313–321.

Pia MV. 2013. Trophic interactions between puma and endemic culpeo fox after livestock removal in the high mountains of central Argentina. mammalia **77**:273–283.

Pokines JT. 2007. Prey Remains from a Great Horned Owl (*Bubo virginianus*) Roost in the Icla Valley, Bolivia. Journal of Raptor Research **41**:174–175.

Pruett-Jones SG, White CM, Devine WR. 1980. Breeding of the peregrine falcon in Victoria, Australia. Emu **80**:253–269.

Purdey DC, King CM, Lawrence B. 2004. Age structure, dispersion and diet of a population of stoats (*Mustela erminea*) in southern Fiordland during the decline phase of the beech mast cycle. New Zealand Journal of Zoology **31**:205–225.

Rau JR, Jiménez JE. 2002. Diet of puma (*Puma concolor*, Carnivora: Felidae) in coastal and Andean ranges of southern Chile. Studies on Neotropical Fauna and Environment **37**:201–205.

Rickard CG. 1996. Introduced small mammals and invertebrate conservation in a lowland podocarp forest, South Westland, New Zealand. Available from http://ir.canterbury.ac.nz/handle/10092/6182 (accessed February 23, 2016).

Robertshaw JD, Harden RH. 1985. The Ecology of the Dingo in North-Eastern New South Wales. 2. Diet. Wildlife Research **12**:39–50.

Robertshaw JD, Harden RH. 1986. The Ecology of the Dingo in Northeastern New-South-Wales. 4. Prey Selection by Dingoes, and Its Effect on the Major Prey Species, the Swamp Wallaby, Wallabia-Bicolor (Desmarest). Wildlife Research **13**:141–163.

Astorga Saavedra DV. 2013. Comparación de la dieta de dos carnívoros silvestres, Güiña (*Leopardus guigna*) y zorro chilla (*Pseudalopex griseus*), en el parque nacional nahuelbuta, Región de la Araucanía, Chile. Available from http://cybertesis.uach.cl/tesis/uach/2013/fva858c/doc/fva858c.pdf (accessed February 23, 2016).

Sade S, Rau JR, Orellana JI. 2012. Dieta del quique (*Galictis cuja* Molina 1782) en un remanente de bosque valdiviano fragmentado del sur de Chile. Gayana (Concepción) **76**:112–116.

Saggese MD, De Lucca ER. 2001. Biología reproductiva del Aguila Mora (*Geranoaetus melanoleucus*) en la Patagonia sur, Argentina. Hornero **16**:77–84.

Salvador-Jr LF, Salim L, Pinheiro M, Granzinolli M. 2013. Observations of a nest of the Black-chested Buzzard-eagle *Buteo melanoleucus* (Accipitridae) in a large urban center in southeast Brazil. Revista Brasileira de Ornitologia-Brazilian Journal of Ornithology **16**:6.

Santander FJ, Alvarado SA, Ramírez PA, Figueroa RA. 2011. Prey of Harris' Hawks (*Parabuteo unicinctus*) During Autumn and Winter in a Coastal Area of Central Chile. The Southwestern Naturalist **56**:417–422.

Santillán MA, Travaini A, Fernández J. 2010. Dieta del Halcón Peregrino (*Falco peregrinus*) en la Ría Deseado, Patagonia austral, Argentina. Boletín Chileno de Ornitología **16**:1–8.

Schlatter RP, Yáñez JL, Jaksić FM. 1980. Food-niche relationships between Chilean Eagles and Red-backed Buzzards in central Chile. The Auk:897–898.

Sharp A, Gibson L, Norton M, Marks A, Ryan B, Semeraro L. 2002a. An evaluation of the use of regurgitated pellets and skeletal material to quantify the diet of Wedge-tailed Eagles, Aquila audax. Emu **102**:181–185.

Sharp A, Gibson L, Norton M, Ryan B, Marks A, Semeraro L. 2002b. The breeding season diet of wedge-tailed eagles (*Aquila audax*) in western New South Wales and the influence of Rabbit Calicivirus Disease. Wildlife Research **29**:175–184.

Short J, Calver MC, Risbey DA. 1999. The impact of cats and foxes on the small vertebrate fauna of Heirisson Prong, Western Australia. I. Exploring potential impact using diet analysis. Wildlife Research **26**:621–630.

Silva L M, Croft DB. 2007. Nest-site selection, diet and parental care of the wedge-tailed eagle *Aquila audax* in western new south wales. Available from http://www.researchgate.net/profile/David_Croft2/publication/235973758_Nest-site_selection_diet_and_parental_care_of_the_Wedge-tailed_Eagle_Aquila_audax_in_Western_New_South_Wales/links/00b4951d91d788d793000000.pdf (accessed November 9, 2015).

Skewes O, Moraga CA, Arriagada P, Rau JR. 2012. El jabalí europeo (*Sus scrofa*): Un invasor biológico como presa reciente del puma (*Puma concolor*) en el sur de Chile. Revista chilena de historia natural **85**:227–232.

Smith GP, Ragg JR, Moller H, Waldrup KA. 1995. Diet of feral ferrets (*Mustela furo*) from pastoral habitats in Otago and Southland, New Zealand. New Zealand journal of zoology **22**:363–369.

Starker Leopole A, Wolfe TO. 1970. Food habits of nesting Wedge-tailed Eagles, *Aquila audax*, in south-eastern Australia. Wildlife Research **15**:1–17.

Teta P, Malzof S, Quintana R, Pereira J. 2006. Presas del ñacurutú (*Bubo virginianus*) en el bajo delta del río Paraná (Buenos Aires, Argentina). Ornitología Neotropical **17**:441–444.

Travaini A, Donázar JA, Ceballos O, Hiraldo F. 2001. Food habits of the Crested Caracara (*Caracara plancus*) in the Andean Patagonia: the role of breeding constraints. Journal of Arid Environments **48**:211–219.

Travaini A, Santillán MA, Zapata SC. 2012. Diet of the Red-backed Hawk (*Buteo polyosoma*) in two environmentally contrasting areas of Patagonia. Studies on Neotropical Fauna and Environment **47**:25–32.

Trejo A, Grigera D. 1998. Food habits of the great horned owl (*Bubo virginianus*) in a Patagonian steppe in Argentina. Journal of Raptor Research **32**:306–311.

Trejo A, Guthmann N. 2003. Owl selection on size and sex classes of rodents: activity and microhabitat use of prey. Journal of Mammalogy **84**:652–658.
Trejo A, Kun M, Sahores M, Seijas S. 2005. Diet overlap and prey size of two owls in the forest-steppe ecotone of southern Argentina. Ornitología Neotropical **16**:539–546.
Trejo A, Kun M, Seijas S. 2006a. Dieta del Águila Mora (*Geranoaetus melanoleucus*) en una transecta oeste-este en el ecotono norpatagónico. El hornero **21**:31–36.
Trejo A, Lambertucci S. 2007. Feeding habits of Barn Owls along a vegetative gradient in northern Patagonia. Journal of Raptor Research **41**:277–287.
Trejo A, Ojeda V, Kun M, Seijas S. 2006b. Prey of White-throated Hawks (*Buteo albigula*) in the southern temperate forest of Argentina. Journal of Field Ornithology **77**:13–17.
Valenzuela AE, Rey AR, Fasola L, Samaniego RAS, Schiavini A. 2013. Trophic ecology of a top predator colonizing the southern extreme of South America: Feeding habits of invasive American mink (*Neovison vison*) in Tierra del Fuego. Mammalian Biology-Zeitschrift für Säugetierkunde **78**:104–110.
Vargas RJ, Bó MS, Favero M, Morrison JL. 2007. Diet of the southern caracara (*Caracara plancus*) in mar chiquita reserve, southern argentina. Journal of Raptor Research **41**:113–121.
Walker RS, Novaro AJ, Perovic P, Palacios R, Donadio E, Lucherini M, Pia M, López MS. 2007. Diets of three species of Andean carnivores in high-altitude deserts of Argentina. Journal of Mammalogy **88**:519–525.
White EM, Wilson JC, Clarke AR. 2006. Biotic indirect effects: a neglected concept in invasion biology. Diversity and Distributions **12**:443–455.
Whitehouse SJO. 1977. The diet of the dingo in Western Australia. Wildlife Research **4**:145–150.
Yáñez JL, Cárdenas JC, Gezelle P, Jaksić FM. 1986. Food habits of the southernmost mountain lions (*Felis concolor*) in South America: natural versus livestocked ranges. Journal of Mammalogy:604–606.
Zapata SC, Travaini A, Delibes M, Martínez-Peck R. 2005. Food habits and resource partitioning between grey and culpeo foxes in southeastern Argentine Patagonia. Studies on Neotropical Fauna and Environment **40**:97–103.
Zúñiga AH, Muñoz-Pedreros A. 2014. Hábitos alimentarios de Puma concolor (Carnivora, Felidae) en bosques fragmentados del sur de Chile. Mastozoología neotropical **21**:157–161.
Zúñiga A, Muñoz-Pedreros A, Fierro A. 2008. Dieta de *Lycalopex griseus* (Gray, 1837)(Mammalia: Canidae) en la depresión intermedia del sur de Chile. Gayana (Concepción) **72**:113–116.
