# Supplementary material for: Medium-sized exotic prey create novel food webs: the case of predators and scavengers consuming lagomorphs
Source: PeerJ. 2016 Jul 27;4:e2273. doi: 10.7717/peerj.2273 (PMC4974932; doi:10.7717/peerj.2273)
Supplement: Table S2 — Detailed results of the four meta-analyses performed in this study. Estimate is the effect size interpreted as interaction strength. The first four results correspond to the four separated meta-analysis: lagomorphs as prey, most consumed preys in presence of lagomorphs, random preys in presence of lagomorphs and the most consumed preys in absence of lagomorphs. The results for each moderator variables correspond to the analysis of lagomorphs as exotic preys in the diet of the predators. Signif. codes p-value: ‘∗∗∗′ = 0 > p > 0.001, ‘∗∗′ = 0.001 > p > 0.01, ‘∗′ = 0.01 > p > 0.05, ‘.′ = 0.05 > p > 0.1, ‘′ = p > 0.1. [file peerj-04-2273-s007.pdf]

Supporting information for

Medium-sized exotic prey create novel food webs: the case of predators and scavengers consuming lagomorphs

Authors

Facundo Barbar[1], Fernando Hiraldo[2], Sergio A. Lambertucci[1].

Authors' affiliations

[1] Grupo de Biología de la Conservación, Laboratorio Ecotono INIBIOMA (CONICET- Universidad Nacional del Comahue).

[2] Departamento de Biología de la Conservación, Estación Biológica Doñana (CSIC) Sevilla, España.

Table S2. Detailed results of the four meta-analyses performed in this study. Estimate is the effect size interpreted as interaction strength. The first four results correspond to the four separated meta-analysis: lagomorphs as prey, most consumed preys in presence of lagomorphs, random preys in presence of lagomorphs and the most consumed preys in absence of lagomorphs. The results for each moderator variables correspond to the analysis of lagomorphs as exotic preys in the diet of the predators. Signif. codes *p*-value: '***'=0> *p* >0.001, '**'=0.001> *p* >0.01, '*'=0.01> *p* >0.05, '.'= 0.05> *p* >0.1, ' '= *p*>0.1.

Results for the 4 separated meta-analysis performed with each item prey class

	estimate	se	zval	pval	ci.lb	ci.ub	
**Lagomorphs**	0.2047	0.0146	14.0487	<.0001	0.1762	0.2333	***

	estimate	se	zval	pval	ci.lb	ci.ub	
**Native w Lagomorphs**	0.1649	0.0133	12.4167	<.0001	0.1389	0.1909	***

	estimate	se	zval	pval	ci.lb	ci.ub
**Native w/o Lagomorphs**	0.2391	0.0320	7.4790	<.0001	0.1764	0.3017

	estimate	se	zval	pval	ci.lb	ci.ub	
**Random Prey**	0.0373	0.0062	5.9985	<.0001	0.0251	0.0495	***

Results for the lagomorphs and predator interaction separated with moderator variables

Moderator variable= lagomorph species consumed

	estimate	se	zval	pval	ci.lb	ci.ub	
Lepus europaeus	0.1752	0.0229	7.6558	<.0001	0.1304	0.2201	***
Oryctolagus cuniculus	0.2245	0.0188	11.9686	<.0001	0.1877	0.2613	***

Moderator variable= Predator species consuming Lagomorphs

	estimate	se	zval	pval	ci.lb	ci.ub	
Accipiter fasciatus	0.3000	0.0836	3.5877	0.0003	0.1361	0.4639	***
Aquila audax	0.3049	0.0385	7.9260	<.0001	0.2295	0.3803	***
Asio flammeus	0.6071	0.2040	2.9762	0.0029	0.2073	1.0070	**
Athene cunicularia	0.0010	0.1819	0.0054	0.9957	-0.3556	0.3576	
Bubo magellanicus	0.0748	0.0551	1.3576	0.1746	-0.0332	0.1829	
Buteo albigula	0.0017	0.1819	0.0092	0.9927	-0.3549	0.3583	
Buteo polyosoma	0.1164	0.0756	1.5393	0.1237	-0.0318	0.2647	
Canis lupus dingo	0.1088	0.0609	1.7869	0.0740	0.1050	0.2282	.
Caracara plancus	0.0540	0.1051	0.5144	0.6070	-0.1519	0.2600	
Circus approximans	0.0958	0.1053	0.9099	0.3629	-0.1105	0.3021	
Circus assimilis	0.5239	0.1838	2.8499	0.0044	0.1636	0.8843	**
Circus cinereus	0.0010	0.1286	0.0078	0.9938	-0.2511	0.2531	
Coragyps atratus	0.1318	0.1822	0.7236	0.4963	-0.2252	0.4889	
Dasyurus geofroyii	0.0102	0.1820	0.0561	0.9553	-0.3465	0.3669	
Dasyurus maculatus	0.0783	0.0645	1.2147	0.2245	-0.0480	0.2047	
Falco berigora	0.1864	0.0649	2.8743	0.0040	0.0593	0.3136	**
Falco cenchroides	0.0056	0.1820	0.0310	0.9752	-0.3511	0.3624	
Falco peregrinus	0.0151	0.1054	0.1428	0.8864	-0.1916	0.2217	
Falco subniger	0.4030	0.1839	2.1915	0.0284	0.0426	0.7634	*
Galactis cuja	0.3262	0.1355	2.4080	0.0160	0.0607	0.5917	*
Geranoaetus melanoleucus	0.4277	0.0557	7.6739	<.0001	0.3185	0.5369	***
Haliaeetus leucogaster	0.0345	0.1827	0.1887	0.8503	-0.3236	0.3926	
Haliastur sphenurus	0.2481	0.0833	2.9796	0.0029	0.0849	0.4113	**
Hamirostra melanosternon	0.3930	0.1295	3.0349	0.0029	0.1392	0.6469	**
Hieraaetus morphnoides	0.3252	0.0833	3.9405	<.0001	0.1634	0.4869	***
Leopardus colocolo	0.0505	0.1318	0.3832	0.7016	-0.2078	0.3088	
Leopardus geofroyii	0.2200	0.0920	2.3905	0.0168	0.0396	0.4005	*

Leopardus jacobitus	0.0267	0.1829	0.1458	0.8841	-0.33318	0.3851	
Lycalopex culpaeus	0.2511	0.0464	5.4080	<.0001	0.1601	0.3421	***
Lycalopex fulvipes	0.0043	0.1819	0.0236	0.9812	-0.3523	0.3609	
Lycalopex griseus	0.1443	0.0824	1.7500	0.0801	-0.0173	0.3059	.
Lycalopex gymnocercus	0.1811	0.0783	2.3121	0.0208	0.0276	0.3347	*
Milvago chimango	0.0007	0.1819	0.0036	0.9971	-0.3559	0.3572	
Mustela erminea	0.2029	0.0705	2.8782	0.0040	0.0647	0.3411	**
Mustela furo	0.6168	0.0885	6.9681	<.0001	0.4433	0.7903	***
Mustela nivea	0.1111	0.1964	0.5657	0.5716	-0.2739	0.4961	
Oncifelis guigna	0.1317	0.1337	0.9855	0.3244	-0.1303	0.3938	
Parabuteo unicinctus	0.0784	0.0748	1.0485	0.2944	-0.0682	0.2250	
Phalcoboenus australis	0.1153	0.1360	0.8481	0.3964	-0.1512	0.3818	
Puma concolor	0.2702	0.0512	5.2797	<.0001	0.1699	0.3705	***
Tyto alba	0.0101	0.0910	0.1105	0.9120	-0.1684	0.1885	
Vulpes vulpes	0.1537	0.0432	3.5591	0.0004	0.0691	0.2384	***
Vultur gryphus	0.2360	0.1829	1.2903	0.1969	-0.1225	0.5944	

Moderator variable= Family of predators consuming Lagomorphs

	estimate	se	zval	Pval	ci.lb	ci.ub	
**Accipitridae**	0.2657	0.0238	11.1546	<.0001	0.2190	0.3124	***
**Canidae**	0.1741	0.0271	6.4212	<.0001	0.1210	0.2273	***
**Cathartidae**	0.1837	0.1406	1.3064	0.1914	-0.0919	0.4594	
**Dasyuridae**	0.0707	0.0662	1.0680	0.2855	-0.0591	0.2006	
**Falconidae**	0.1229	0.0459	2.6776	0.0074	0.0329	0.2129	**
**Felidae**	0.2182	0.0459	5.0883	<.0001	0.1341	0.3022	***
**Mustelidae**	0.3427	0.0536	6.3986	<.0001	0.2377	0.4477	***
**Strigidae**	0.0982	0.0535	1.8335	0.0667	-0.0068	0.2031	.
**Tytonidae**	0.0035	0.1145	0.0306	0.9756	-0.2209	0.2279	

Moderator variable= Class of predator consuming Lagomorphs

	estimate	se	zval	pval	ci.lb	ci.ub	
**Birds**	0.2124	0.0207	10.2559	<.0001	0.1718	0.253	***
**Mammals**	0.1956	0.0289	6.7685	<.0001	0.1389	0.2522	***

Moderator variable= Region of each study retrieved where native predators consume Lagomorphs

	estimate	se	zval	pval	ci.lb	ci.ub	
**Oceania**	0.2233	0.0205	10.9158	<.0001	0.1832	0.2634	***
**Southamerica**	0.1857	0.0207	8.9773	<.0001	0.1452	0.2263	***

Moderator variable= Region of each study retrieved where native predators consume Lagomorphs

	estimate	se	zval	pval	ci.lb	ci.ub	
**Native**	0.2052	0.0162	12.6497	<.0001	0.1734	0.2370	***
**Exotic**	0.2028	0.0336	6.0360	<.0001	0.1370	0.2687	***
